# Lung function associated gene Integrator Complex subunit 12 regulates protein synthesis pathways

**DOI:** 10.1186/s12864-017-3628-3

**Published:** 2017-03-23

**Authors:** Alexander K. Kheirallah, Cornelia H. de Moor, Alen Faiz, Ian Sayers, Ian P. Hall

**Affiliations:** 1 Wellcome Trust - Medical Research Council Cambridge Stem Cell Institute, University of Cambridge, Cambridge, UK; 2 Division of Respiratory Medicine, School of Medicine, University of Nottingham, Nottingham, NG7 2UH UK; 3 Division of Molecular and Cellular Sciences, School of Pharmacy, University of Nottingham, Nottingham, NG7 2RD UK; 4 Department of Pulmonology, University of Groningen, Groningen, 9713 GZ Netherlands

**Keywords:** Integrator Complex, INTS12, snRNA processing, Protein synthesis, Regulation of gene expression, Pathway dysregulation, Histone modification, Accessible chromatin, Transcription

## Abstract

**Background:**

Genetic studies of human lung function and Chronic Obstructive Pulmonary Disease have identified a highly significant and reproducible signal on 4q24. It remains unclear which of the two candidate genes within this locus may regulate lung function: *GSTCD*, a gene with unknown function, and/or *INTS12*, a member of the Integrator Complex which is currently thought to mediate 3’end processing of small nuclear RNAs.

**Results:**

We found that, in lung tissue, 4q24 polymorphisms associated with lung function correlate with *INTS12* but not neighbouring *GSTCD* expression. In contrast to the previous reports in other species, we only observed a minor alteration of snRNA processing following INTS12 depletion. RNAseq analysis of knockdown cells instead revealed dysregulation of a core subset of genes relevant to airway biology and a robust downregulation of protein synthesis pathways. Consistent with this, protein translation was decreased in INTS12 knockdown cells. In addition, ChIPseq experiments demonstrated INTS12 binding throughout the genome, which was enriched in transcriptionally active regions. Finally, we defined the INTS12 regulome which includes genes belonging to the protein synthesis pathways.

**Conclusion:**

INTS12 has functions beyond the canonical snRNA processing. We show that it regulates translation by regulating the expression of genes belonging to protein synthesis pathways. This study provides a detailed analysis of INTS12 activities on a genome-wide scale and contributes to the biology behind the genetic association for lung function at 4q24.

**Electronic supplementary material:**

The online version of this article (doi:10.1186/s12864-017-3628-3) contains supplementary material, which is available to authorized users.

## Background

According to the World Health Organization, respiratory diseases such as Chronic Obstructive Pulmonary Disease (COPD) are one of the leading causes of population morbidity and mortality [1]. COPD is characterized by irreversible airway obstruction, and one or both of emphysema and chronic bronchitis. Clinically, COPD is defined by lung function parameters, the forced vital capacity and the forced expiratory volume in the first second [2]. Since the beginning of genome-wide association studies (GWAS), efforts were undertaken to further our understanding of the pathobiology of this disease. Several studies have identified highly significant associations between single nucleotide polymorphisms (SNPs) on chromosome 4q24 and lung function as well as risk of COPD [3–6]. However, the mechanistic basis of this signal has not been elucidated. To understand the functional basis for this region, we have previously used expression quantitative trait locus (eQTL) analyses in multiple non-lung tissues and found the strongest evidence supported the hypothesis that the variable expression of Integrator Complex subunit 12 gene (*INTS12*) underlies this association [7].

INTS12 protein is a member of Integrator Complex (INTScom) currently believed to be composed of approximately 14 subunits [8]. This complex was shown to stably accompany the C-terminal tail of RNA polymerase II (POLII) and at a molecular level has been implicated in small nuclear RNA (snRNA) biogenesis [9–12] dynein recruitment to the nuclear envelope at the mitotic onset [13] and with POLII pause and release [14]. At the physiological level, targeted knockdown and mutagenesis experiments demonstrated INTScom to be necessary for mouse adipogenesis [15], zebrafish haemopoiesis [16] as well as human primary ciliogenesis [17]. The relative contributions of individual subunits in the above processes vary.

Direct insights into the function of INTS12 come from *Drosophila* where it is necessary for the spliceosome’s snRNA processing and this function is considered to be canonical [10–12]. *Drosophila*’s INTS12 was also implicated in the activation of a key heat shock response gene *HSP70Aa* [14]. In HeLa cells, INTS12 was specifically shown to be required for the maintenance of perinuclear dynein [13] and formation of primary cilia [17]. Although ciliogenesis is a dynein-dependent event [18, 19] it is thought that INTS12 is regulating these two processes separately from each other via the snRNA processing pathway [17]. INTS12 is also likely to play important roles in embryonic development. This has been supported by studies which showed that homozygous *INTS12* knockout in *M. musculus* results in pre-weaning lethality [7]. The lethal effect of *INTS12* knockout most probably occurs *in utero* as breeding heterozygous models only yields wild-type homozygotes or mutant heterozygotes but never produces homozygous litters with no functional copy of the gene (data not shown). In *Drosophila*, the evolutionary conserved INTS12’s plant homeodomain (PHD) is dispensable for the canonical snRNA processing [12] suggesting the probable existence of other functions for this protein. Thus although numerous INTS12 dependent cellular functions have been reported, no studies have addressed by which molecular mechanisms these functions are implemented.

Here we investigate the regulatory properties of INTS12 in primary human bronchial epithelial cells (HBECs) to help understand the biological mechanism behind the association signal for lung function at 4q24 locus. As no genome-wide molecular assessment of INTS12 perturbation has been performed to date, we use a hypothesis-free approach [20] by combining gene knockdown with RNA sequencing in order to generate new functional hypotheses. We bioinformatically show that INTS12 has homology to epigenetic regulators of gene expression. As this molecule was shown to interact with genomic DNA in flies [14], we performed chromatin immunoprecipitation followed by sequencing (ChIPseq) and combined it with RNAseq data. Our data show that INTS12 acts as a regulator of pathways fundamental for protein synthesis, including the tRNA synthetases, PERK and unfolded protein response pathways. We provide insights into the characteristics of INTS12 binding as well as its relationship to transcription and propose a model for INTScom activity that may explain the plethora of phenotypes observed upon depletion of various members of the complex. Finally, we suggest that variation in *INTS12* expression conferred by specific eQTL alleles, dictates the levels of protein synthesis and thus may in part be contributing to the genetic association for lung function.

## Results

### Lung function SNPs are eQTLs for *INTS12* expression in the lung tissue

The association signal for lung function within 4q24 contains a peak situated over two oppositely transcribed genes in close proximity to each other, the Glutathione S-transferase, C-terminal Domain Containing (*GSTCD*) and *INTS12*. Based on eQTL analyses in non-lung tissues, it has been suggested that *INTS12* is a more likely contributor to the pulmonary function than *GSTCD* (Obeidat et al. 2013). To confirm this observation, we have taken advantage of a RNAseq-based human lung eQTL dataset from the Genotype-Tissue Expression project [21]. There were 248 SNPs at or near 4q24 that were significant *cis*-eQTLs for *INTS12* expression (*n* = 278, false discovery rate (FDR) < 0.05). Among these, 30 SNPs showed significant association for lung function in the SpiroMeta consortium study [3]. In contrast, none of these variants showed significant association with *GSTCD* expression (Table 1). This finding indicates that within 4q24 it is the altered expression of *INTS12*, and not *GSTCD*, that is influencing lung function.

**Table 1:** INTS12 *cis*-eQTLs at 4q24 locus

SNP	Position	FEV1 P-value	INTS12 eQTL FDR	INTS12 effect size	GSTCD eQTL FDR	GSTCD effect size
rs11732650	106973680	6.83E-09	0.000397993	−0.53	1	0
rs11722225	106985879	7.08E-09	0.000397993	−0.53	1	0
rs11726124	106985945	6.63E-09	0.000397993	−0.53	1	0
rs11728716	106975445	8.44E-09	0.000397993	−0.53	1	0
rs17036090	106813023	3.84E-08	0.000397993	−0.51	1	0.01
rs11735851	106916703	1.90E-09	0.000397993	−0.51	1	0.02
rs17036225	106929541	3.33E-09	0.000397993	−0.51	1	0.02
rs11736859	106928234	2.86E-09	0.000397993	−0.51	1	0.02
rs11727745	106935976	5.47E-09	0.000397993	−0.51	1	0.02
rs10516528	106959042	6.27E-09	0.000397993	−0.51	1	0.02
rs17036139	106852106	1.25E-09	0.000397993	−0.51	1	0.02
rs11727189	106838589	3.38E-09	0.000397993	−0.51	1	0.02
rs11731417	106965461	5.96E-09	0.000397993	−0.53	1	0
rs11733287	106924788	2.32E-09	0.000397993	−0.53	1	0
rs11728044	106824235	1.95E-09	0.000397993	−0.51	1	0.02
rs11733225	106924812	2.34E-09	0.000397993	−0.51	1	0.02
rs10516525	106887474	1.44E-09	0.000397993	−0.51	1	0.02
rs11724839	106857705	1.79E-09	0.000397993	−0.51	1	0.02
rs10516526	106908353	6.67E-10	0.000397993	−0.51	1	0.02
rs17036142	106854185	1.11E-09	0.000397993	−0.51	1	0.02
rs12374256	106836810	1.88E-09	0.000658031	−0.52	1	0.03
rs11097901	106949382	6.32E-09	0.000953622	−0.47	1	0.02

Genome-wide significant SNPs for lung function parameter forced expiratory volume in the first second (FEV1) also correlate with *INTS12* expression in the human lung (FDR < 0.001). This observation is not true for the expression of neighbouring *GSTCD*, supporting the hypothesis that altered expression of *INTS12* is driving the association signal for lung function. Effect size is defined as the slope of linear regression line relative to reference allele normalized as an expression of 1. The eQTL data was obtained from Genotype-Tissue Expression project, while per SNPs lung function significance values in linkage disequilibrium with INTS12 (r^2^ > 0.8) are from Repapi et al. study (Repapi et al. 2010)[3]

### Human INTS12 knockdown has modest effects on snRNA processing in HBECs

**Fig. 1:**
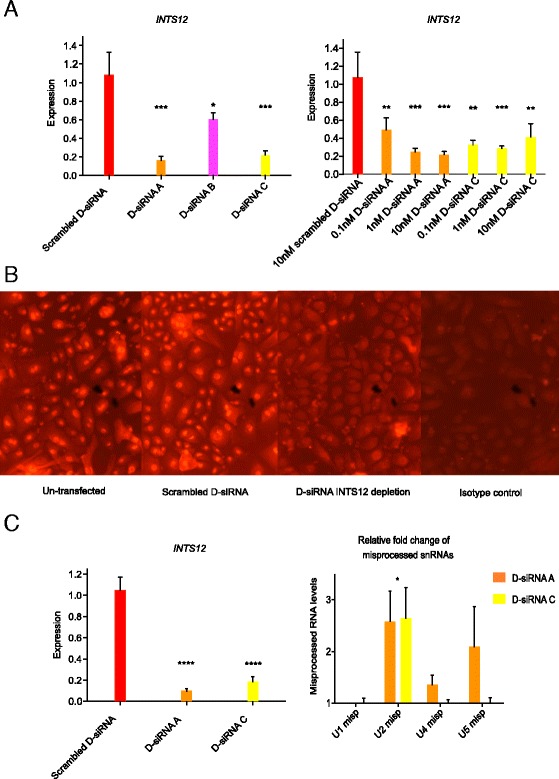
Optimizing INTS12 knockdown and elucidating its effect on snRNA processing in HBECs. **a** Optimizing anti-INTS12 D-siRNA transfections. *INTS12* mRNA expression in HBECs transfected with three distinct D-siRNAs at 10nM (left) and with the indicated concentrations of D-siRNA A and C (right). D-siRNAs A and C at a concentration of 1nM were chosen for subsequent experiments. Statistical tests were performed comparing to scrambled D-siRNA control: *P < 0.05, **P < 0.01, ***P < 0.001. Individual ∆∆Ct gene expressions are *GAPDH* normalized and relative to the mean of scrambled D-siRNA condition. **b** Representative images of INTS12 protein expression in anti-INTS12 D-siRNA transfected HBECs by immunofluorescence. **c** *INTS12* mRNA expression in HBECs transfected with D-siRNA A and C (left) and corresponding fold changes in misprocessed snRNAs (right). Statistical tests were performed comparing to scrambled D-siRNA control: *P < 0.05, ****P < 0.0001. Individual ∆∆Ct gene expressions are *GAPDH* normalized and relative to the mean of scrambled D-siRNA condition

Given previous observations in *Drosophila* implying a role for *INTS12* in processing of U1, U2, U4 and U5 snRNAs [10–12], we first set out to determine if these observations translate to a human model. Because *INTS12* expression is higher in the human bronchial epithelium than other airway structural cells [7] we concentrated our studies on primary HBECs grown to passage three. Quantitative PCR (qPCR) assays measuring misprocessed U1, U2, U4 and U5 snRNAs were developed. We then validated transfection conditions and demonstrated knockdown, both at the mRNA (Fig. 1a) and protein levels (Fig. 1b, Additional file 1: Figure S1), with two different Dicer substrate siRNAs (D-siRNAs) [22] targeting INTS12.

Transfection of primary cultures of HBECs with D-siRNAs A and C produced 91 ± 2% and 82 ± 3% knockdown of INTS12, respectively (Fig. 1c). In contrast to findings in *Drosophila*, no significant effects on U1 processing were seen. A role for INTS12 on U2 processing was found, with fold increases in U2 immature product by 2.58 ± 0.58 and by 2.64 ± 0.59 for D-siRNAs A and C respectively (*P* < 0.05; Fig. 1c). However, in keeping with the lack of impact on U1 processing, we found no significant effects of INTS12 knockdown on processing of U4 and U5 snRNAs. These data suggest that whilst INTS12 may play a role in U2 processing, it does not significantly affect processing of U1, U4, and U5 snRNAs in HBECs. Due to ubiquitous expression of snRNA genes [9–11], we cannot exclude the possibility that effects on other snRNA species were not observed due to the requirement for a more robust INTS12 protein knockdown than was achieved. However, a survey of published studies that investigated the importance of INTS12, indicates a potentially weaker role for this molecule in delivering snRNA processing relative to other INTScom members (Additional file 2: Table S6).

**Fig. 2:**
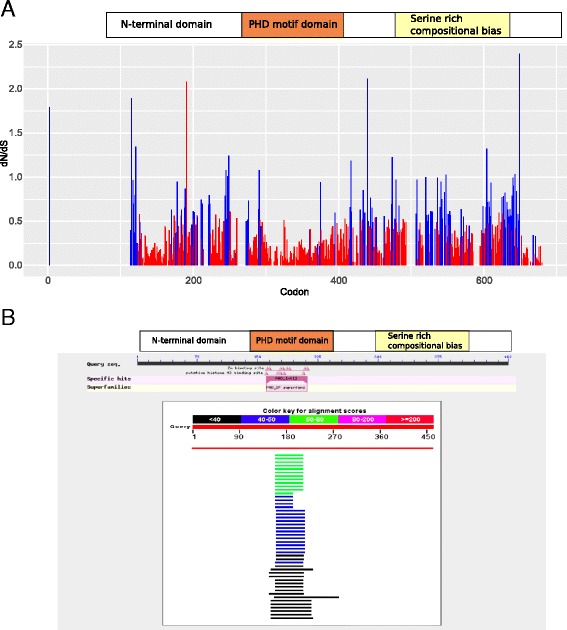
Sequence analysis of INTS12’s PHD and its sequence similarity to epigenetic regulators of gene expression. **a** Quantitative assessment of INTS12 conservation using a repertoire of 66 metazoan open reading frames. The ratio of non-synonymous changes to synonymous changes (dN/dS) is shown throughout the protein. dN/dS approaching zero indicate strong and significant conservation (*n* = 66 species): red colour *P* < 0.1, blue colour *P* > 0.1. P-value represents the probability of observed dN/dS ratio given the null the hypothesis of neutral evolution. **b** Full length INTS12 protein sequence (NP_001135943.1) BLASTP against a database of *Homo sapiens* protein sequences shows the sequence similarity to be exclusively within the PHD. PHD appears as a putative zinc and histone H3 binding site

Sequence analysis of open reading frames (ORF) from 66 metazoan species revealed high levels of INTS12 conservation, particularly its PHD (Fig. 2a). The observed effects on snRNA processing together with the detected conservation prompted us to hypothesize the existence of additional functions for INTS12. Moreover, the evolutionary constrained PHD finger is dispensable for snRNA processing in *Drosophila* [12]. In order to gain insight into the potential INTS12 functions, a search of similar human proteins was performed using the BLASTP algorithm [23] (Fig. 2b). INTS12’s PHD appeared to be homologous to a large family of fingers whose functions lie in the control of chromatin and nucleosomes [24] where they act as epigenetic regulators of gene expression (Additional file 2: Table S1 and S2). Therefore, we next aimed to study the genome-wide regulatory properties of INTS12 by using a combination of gene knockdown with transcriptome profiling and patterns of binding to the genomic DNA.

### Differential transcriptome analysis reveals regulation of a core regulome subset of relevance to airway biology

**Fig. 3:**
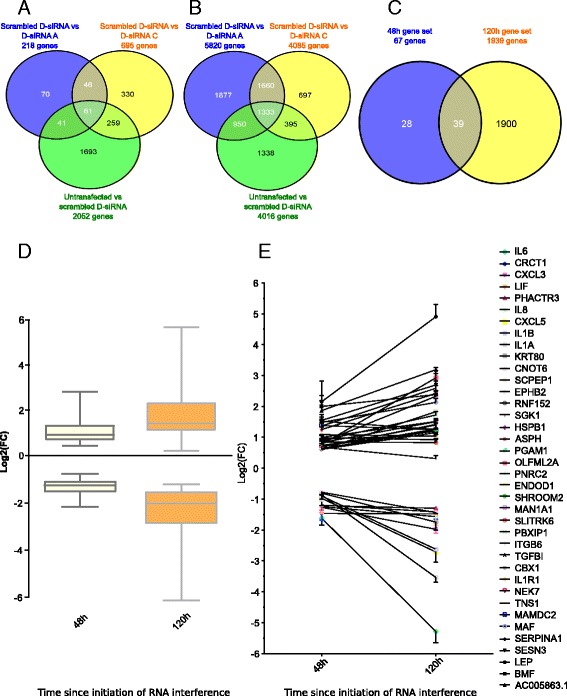
Differential transcriptome analysis reveals regulation of a core subset of genes relevant to airway biology. Statistical tests were performed comparing to scrambled D-siRNA control: all significantly deregulated genes had FDR < 0.05. **a** Venn diagrams of significantly deregulated genes at 48 h in the indicated conditions. 46 reproducibly deregulated genes plus 21 out of 61 genes deregulated in all three comparisons but in opposite direction in INTS12 knockdown conditions when compared to un-transfected vs. scrambled D-siRNA analysis were shortlisted from 48 h dataset (total 67). **b** Venn diagrams of significantly deregulated genes at 120 h. 1660 reproducibly deregulated genes plus 279 out of 1333 genes deregulated in all three comparisons but in opposite direction in INTS12 knockdown conditions when compared to un-transfected vs. scrambled D-siRNA analysis were shortlisted from 120 h dataset (total 1939). **c** Comparison of 48 h and 120 h transcriptomic responses to INTS12 knockdown. The two gene sets contain 39 common with overrepresentation of genes of relevance to airway biology. **d** Box plot of log_2_ fold changes in gene expression observed in 48 h and 120 h transcriptomic responses to gene knockdown using D-siRNA A. Sustained depletion resulted in greater fold changes of gene expression. **e** Log_2_ fold changes of 39 common genes significantly deregulated at 48 h and 120 h using D-siRNA A. Genes have greater effect sizes in 120 h response for all except one

In order to identify a core subset of genes that are significantly regulated by INTS12 we compared the acute versus longer term transcriptomic responses due to depletion. RNAseq profiling was performed 48 h and 120 h after RNA interference (RNAi). After 48 h the levels of knockdown were 74 ± 1% and 78 ± 2%, whilst after 120 h, 89 ± 1% and 80 ± 2% for D-siRNAs A and C respectively (FDR < 0.05). After accounting for off-target and transfection effects there were 67 and 1939 differentially expressed genes by INTS12 knockdown at 48 h and 120 h time points respectively (FDR < 0.05; Fig. 3a and b). Thus, sustained knockdown resulted in a differential expression of ~30 times more genes than what was observed in acute response to knockdown (Fig. 3c). For those genes showing altered levels at both time points, called core regulome subset, the magnitude of change was greater at 120 h post initiation of RNAi (Fig. 3d) for all except one (Fig. 3e). Crucially the direction of differential expression for this set of genes is the same in the independent D-siRNAs treatments and at both time points (Fig. 3e, Table 2).

**Table 2:** Deregulation of a core regulome genes due to INTS12 knockdown

48 h and 120 h consensus genes	FOLD CHANGES			
	48 h		120 h	
	Scrambled vs D-siRNA A	Scrambled vs D-siRNA C	Scrambled vs D-siRNA A	Scrambled vs D-siRNA C
LEP	4.51	16.62	29.16	23.41
AC005863.1	3.35	3.90	9.60	5.55
OLFML2A	1.71	2.55	8.02	2.71
SESN3	2.98	2.01	6.80	2.53
TNS1	2.66	4.73	6.43	6.12
NEK7	2.42	2.30	5.67	3.95
MAN1A1	1.82	2.03	5.26	2.67
MAF	2.81	4.89	4.56	5.47
BMF	3.81	3.48	4.49	4.49
SCPEP1	1.53	1.56	3.76	1.26
PBXIP1	1.88	2.14	3.49	2.32
CBX1	2.04	2.28	3.01	3.39
ENDOD1	1.80	1.82	2.99	3.04
SGK1	1.63	1.54	2.89	1.93
HSPB1	1.65	1.47	2.75	1.48
RNF152	1.55	1.84	2.55	1.97
SERPINA1	2.85	2.71	2.48	2.79
PGAM1	1.64	1.70	2.47	2.02
ASPH	1.59	1.66	2.44	2.37
MAMDC2	2.56	3.17	2.43	7.40
SHROOM2	1.66	1.95	2.42	1.80
EPHB2	1.55	2.12	2.20	2.63
ITGB6	1.84	2.47	2.19	4.01
IL1R1	2.16	2.02	2.16	1.66
TGFBI	1.99	2.75	2.00	5.83
SLITRK6	1.80	2.11	1.86	2.62
PNRC2	1.63	1.56	1.31	1.36
PHACTR3	0.43	0.53	0.43	0.52
IL8	0.45	0.49	0.42	0.18
CRCT1	0.34	0.54	0.39	0.58
CNOT6	0.59	0.64	0.39	0.56
LIF	0.43	0.41	0.38	0.35
KRT80	0.58	0.38	0.32	0.33
CXCL3	0.43	0.35	0.28	0.20
IL1B	0.52	0.38	0.17	0.33
CXCL5	0.54	0.47	0.17	0.34
IL1A	0.55	0.65	0.09	0.49
IL6	0.33	0.48	0.03	0.20

The table is showing the fold changes of consensus differentially expressed genes after 48 h and 120 h since the D-siRNA A and C transfections

Genes showing altered expression include a number of genes known to play important roles in lung disease such as α1-antitrypsin (*SERPINA1*) [25], transforming growth factor β 1 (*TGFβI*) [26], interleukin 1 receptor 1 (*IL1R1*) [27] and *IL6*, *IL8*, *IL1B*, *IL1A* [28–31]. We have calculated the P-value of observing such an association of “lung biology genes” with the list of core regulome assuming the null hypothesis of their independence given the background of protein coding genes. Surprisingly in the light of global effects of INTS12 depletion (Fig. 3b), this analysis allowed us to reject the null hypothesis in favour of the alternative (*P* < 0.0001). *IL6* had the greatest reduction in expression. The gene with the greatest fold induction was Leptin (*LEP*) which was shown to be upregulated and secreted from HBECs infected with respiratory syncytial virus [32] (Table 2). Interestingly, several polymorphisms in linkage with *LEP* are associated with lung function [33]. LEP blood concentration was also shown to negatively correlate with lung function [34]. Crucially, we have biologically validated LEP upregulation in an additional donor HBECs depleted of INTS12 (Additional file 1: Figure S2). These findings suggest that altered expression of *INTS12* in population studies may at least in part contribute to lung biology as well as, more broadly, potentially towards other phenotypes.

In relation to the above findings it is of interest whether INTS12 can work independently from the rest of the INTScom complex or if it mediates tissue-specific functions via this complex. To begin to address this question we have performed a correlation analysis of known INTScom members using our entire 48 h and 120 h expression RNA-seq datasets. It appeared that average Pearson’s correlation coefficients are −0.13 and 0.22 at 48 h and 120 h respectively for INTS12 mRNA expression in relation to the other INTS proteins (Additional file 1: Figure S12). Therefore, in HBECs, INTS12 seems to be expressed independently from the rest of INTScom members and potentially operating independently.

### Differential pathway analysis identifies dysregulation of protein synthesis and collagen formation pathways following INTS12 knockdown

RNAseq transcriptomic profiling of cells depleted of INTS12 for 120 hours was used to generate novel functional hypotheses, because of improved silencing and greater number of expression changes observed at this time point (Fig. 3c). To identify pathways dysregulated as a result of knockdown, Gene Set Enrichment Analysis (GSEA) [35] was used leveraging 4722 curated gene sets from the Molecular Signatures Database which included 1320 canonical pathway definitions [36–38]. To provide internal replication and account for off-target effects [39], we performed GSEA analyses separately following treatment with either D-siRNA A or C, comparing scrambled D-siRNA treated cells to INTS12 depleted cells. Additionally, un-transfected cells were compared with scrambled D-siRNA treated cells to account for pathways that may be altered following treatment with non-specific D-siRNA as artefacts of the experimental exposure rather than being causally related to the gene knockdown. Pathways reproducibly perturbed by both D-siRNAs (FDR < 0.05) but not affected by scrambled D-siRNA treatment were shortlisted and finally top dysregulated pathways were identified based on enrichment score ordering.

**Fig. 4:**
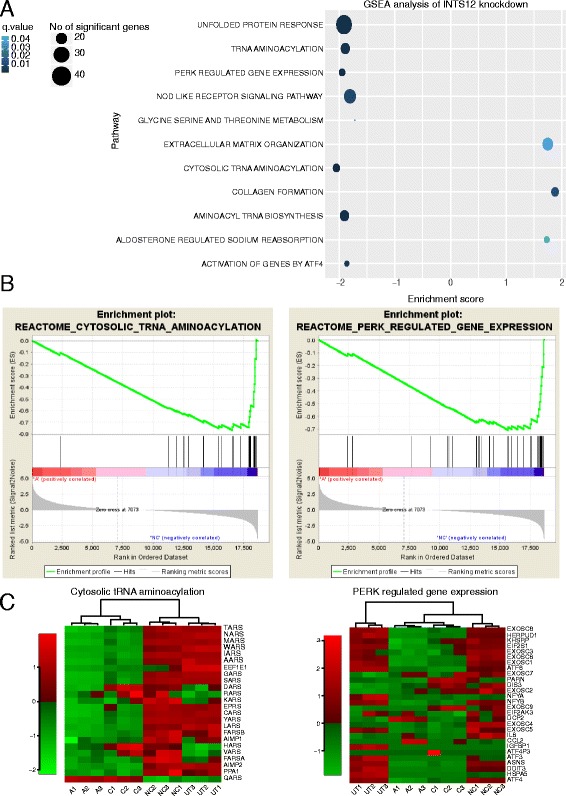
Systematic analysis of dysregulated pathways in INTS12 depleted cells. **a** Cleveland’s plot showing the GSEA results of representative D-siRNA A analysis. Only pathways reproducibly dysregulated in both D-siRNA treatments and not dysregulated by non-specific D-siRNA control treatment are included. The shade of colour indicates statistical significance of enrichment after multiple hypotheses testing correction. The size of dot reflects the number of statistically significant differentially expressed gene. The location of dot reflects enrichment score in pathway analysis. **b** Enrichment plots of cytosolic tRNA aminoacylation and PERK regulated gene expression pathways in D-siRNA A analysis. The FDR values were 0.0004 and 0.002 while normalized enrichment scores were −2.05 and −1.95 for tRNA aminoacylation and PERK regulated gene expression respectively. **c** Heatmaps of genes belonging to tRNA aminoacylation and PERK regulated gene expression pathways. Samples were clustered by unsupervised hierarchical clustering and resulted in clustering of three biological replicate samples of each of the four conditions: un-transfected cells (UT), cells transfected with scrambled D-siRNA negative control (NC), cells transfected with anti-INTS12 D-siRNA A (A) and cells transfected with anti-INTS12 D-siRNA C (C). Green and red colours on the Z-scale indicate lower and higher expression respectively

Using this method three pathways were upregulated and eight pathways were downregulated (Fig. 4a). Collagen formation and extracellular matrix organization pathways were the top two upregulated pathways (Fig. 4a, Additional file 1: Figure S3, Figure S4, Figure S5). The top two downregulated pathways were cytosolic tRNA aminoacylation and PERK regulated gene expression (Fig. 4b, Fig. 4c) which is a sub-pathway of the unfolded protein response (Additional file 1: Figure S3, Figure S6, Figure S7). We also observed significant downregulation of other protein metabolism related pathways, including activation of genes by activating transcription factor 4 (*ATF4*) and glycine, serine and threonine metabolism pathways (Fig. 4a, Additional file 1: Figure S6, Figure S7). *ATF4* expression was reduced by 70 ± 5% and 45 ± 2% in D-siRNA A and C transfected cells when compared to scrambled D-siRNA transfected cells respectively (FDR < 0.05) suggesting an impact on integrated stress response [40]. Dysregulation of these pathways was not observed when comparing un-transfected cells to scrambled D-siRNA cells (Fig. 4c). Overall, we observed greater number of dysregulated gene sets meeting the statistical significance (Fig. 4a), larger effect sizes (Additional file 1: Figure S3), higher number of genes contributing to enrichment score (i.e. leading edge group), greater enrichment scores (Fig. 4B, Additional file 1: Figure S4, Figure S6) and lower variance of gene expression (Fig. 4c, Additional file 1: Figure S5, Figure S7) among the downregulated pathways. This suggests that INTS12 knockdown predominantly results in gene and pathway downregulation. Although these experiments cannot specify whether mechanistically these effects are directly or indirectly caused by INTS12, it is possible to say that they were initiated by INTS12 knockdown and thus may be causally attributed to the levels of this gene.

### INTS12 is a regulator of protein synthesis and proliferative capacity

**Fig. 5:**
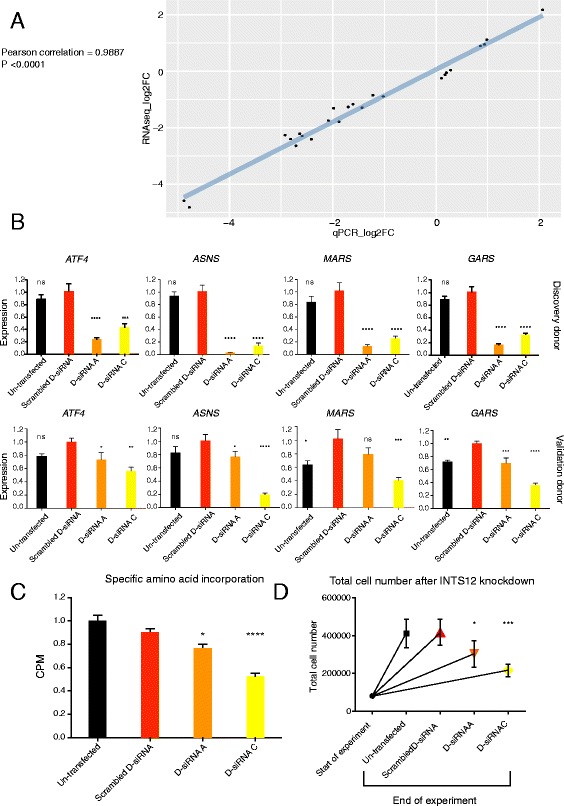
Technical, biological and phenotypic validation of the INTS12 knockdown impact on the protein synthesis pathways. **a** Technical validation of RNAseq findings by qPCR. Differences in gene expression derived from RNAseq strongly and significantly correlate with differences in gene expression derived from qPCR. Validation assays were performed on the same samples that were used for RNAseq study. **b** Biological validation of downregulation of genes belonging to cytosolic tRNA aminoacylation and PERK pathways in HBECs from the discovery donor (used in RNAseq) and in an additional donor (validation donor). Statistical tests were performed comparing to scrambled D-siRNA control: *P < 0.05, **P < 0.01, ***P < 0.001, ****P < 0.0001. Individual ∆∆Ct gene expressions are *GAPDH* normalized and relative to the mean of the scrambled D-siRNA condition. **c** Amino acid incorporation measured by counts per methionine (CPM) in 120 h since the start of RNAi radiolabelling experiment. Statistical tests were performed comparing to scrambled D-siRNA control: *P < 0.05, ***P < 0.001. Individual CPM values are normalized to the amount of total protein and are shown as relative to the mean of the un-transfected condition. **d** HBEC counts at the beginning and at the end of 120 h INTS12 knockdown experiment. Statistical tests were performed comparing to scrambled D-siRNA control: *P < 0.05, ***P < 0.001

In order to validate the RNAseq data, the expression of methionyl-tRNA synthetase (*MARS*) and glycyl-tRNA synthetase (*GARS*) genes from the tRNA synthetases pathway and *ATF4* and Asparagine Synthetase (*ASNS*) genes from the PERK pathway were assessed by qPCR. Analysis revealed the correlation of differences in gene expression derived from RNAseq and qPCR estimates to be 0.99 (Fig. 5a). The effect of knockdown on genes belonging to these top two downregulated pathways was confirmed by independent experiments in cells from an additional donor. Importantly INTS12 was suppressed by 72 ± 4% and 86 ± 2% in the validation donor for D-siRNA A and C respectively versus 93 ± 1% and 85 ± 2% in the discovery donor for D-siRNA A and C respectively which was mirrored by magnitude of changes observed among the assayed target genes (Fig. 5b). These data further support the regulatory effect of INTS12 upon protein translation pathways.

Because of INTS12 knockdown induced downregulation of several key pathways involved in protein metabolism and translational control, the question was whether this manipulation would affect cellular translation phenotype. As predicted, INTS12 silencing repressed protein synthesis by 23 ± 3% and 47 ± 3% in D-siRNA A and C respectively (Fig. 5c). Since cell division requires doubling of protein content prior to separation, we also conjectured that INTS12 depletion would affect the cells’ capacity to proliferate. Interestingly, counts revealed 25 ± 13% and 48 ± 4% decrease of total cell numbers in D-siRNAs A and C conditions respectively (Fig. 5d), mirroring the observed reduction in protein synthesis. Thus the observed molecular signature impacted the relevant phenotypes, demonstrating INTS12 as a regulator of genes forming part of translational pathways.

### Characterization of INTS12 binding sites and their association with fixed elements

The observed regulation of protein synthesis genes could be either indirect, e.g. through disruption of cell homeostasis, or more direct, e.g. via control of gene transcription or a post-transcriptional mechanism. It is not clear which of these scenarios is the case. Therefore, we aimed to test the hypothesis that its binding might be enriched for promoters of genes differentially expressed following knockdown by performing ChIPseq [41] using two independent donors’ HBECs and an antibody that we tested to be specific for INTS12 (Additional file 1: Figure S1). We also intended to investigate INTS12 interaction with both fixed features [42] and cell-type-specific regulatory elements of the human genome [43, 44]. Out of a total of 37142070, 47776470 and 42932683 reads, 78.3%, 78.4 and 77.4% were uniquely mappable, while 81.9%, 83.0 and 93.0% were non-redundant in the first, second donor and isotype control respectively.

We first tested inter-donor reproducibility of the ChIPseq signal. Peak calling revealed that there were 70772 and 51377 binding sites in the first and second donor respectively (FDR < 0.05). An inter-donor association test of ChIPseq signal in active regions demonstrated a significant correlation of 0.85 implying strong biological reproducibility (Additional file 1: Figure S8). In order to verify sequencing results, three positive sites and one negative site were selected for further validation by ChIP-PCR in each ChIP sample. The number of binding events per thousand cells derived from ChIP-PCR corresponded well with the observed ChIPseq signal validating our sequencing results (Additional file 1: Figure S9).

**Fig. 6:**
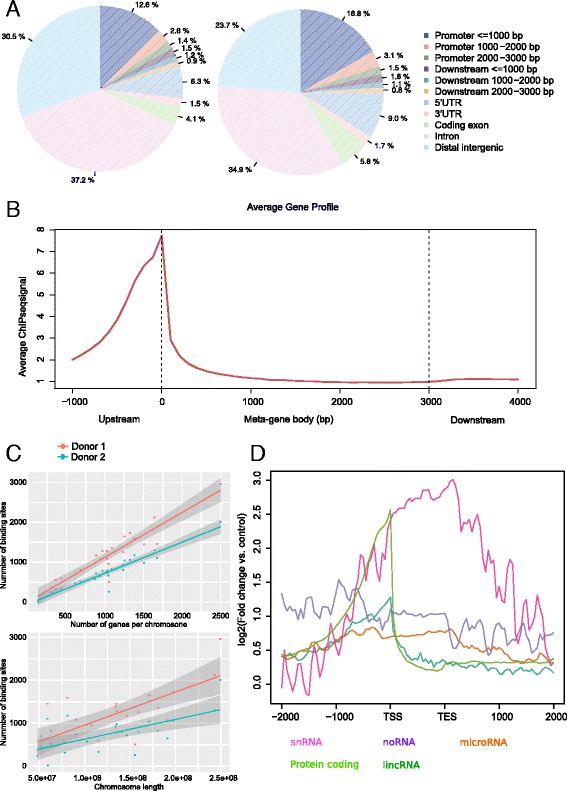
Summary of INTS12 binding to fixed features of the human genome. **a** Percentage of INTS12 binding sites falling on the fixed annotated genomic features in the first (left) and second (right) donor. **b** Gene-centric analysis of INTS12 binding in the first donor across the gene bodies of all the known human genes shows clear localization near the TSS. **c** Relationship of INTS12 binding to the gene number per each chromosome and chromosome length. Analysis of binding versus number of genes revealed Pearson’s correlations of 0.93 and 0.95 in the first and second donor respectively. Instead, correlations of binding sites and chromosome length are weaker being 0.73 and 0.63 for the first and second donor respectively. **d** Comparison of INTS12 binding in the first donor across the bodies of protein coding, snRNA, snoRNA, lincRNA, and microRNA genes

The top three fixed genomic features associated with INTS12 binding were intron, intergenic and promoter (transcriptional start site (TSS) ± 3000 bp) regions. In the first donor they occupied 37.2%, 30.5 and 16.8%, while in the second donor they intersected with 34.9%, 23.7 and 21.4% of the total binding sites respectively (Fig. 6a). We noted that 74.9 and 78.5% of all promoter binding occurred proximally to TSS in the first and second donor respectively. In agreement, a gene-centric analysis over a meta-gene body (collection of hg19 RefSeq genes), revealed INTS12 binding to be in close proximity to the TSS (Fig. 6b).

Next we performed a correlation analysis of INTS12 binding sites with the number of annotated genes and with the nucleotide length of each chromosome. INTS12 binding in both donors correlated very well with the number of genes (Fig. 6c). Correlations with chromosome length were notably weaker (Fig. 6c), indicating that INTS12 is more likely to regulate gene expression rather than being ‘merely distributed’ across the chromosomes. Based on this analysis we conclude that INTS12 binding sites along the genome are closely correlated with genes.

Since the canonical function of INTS12 is processing of snRNAs [9] our initial prediction was that it would be primarily enriched over the bodies of snRNA genes and less so for other gene classes. However, our observation of the widespread distribution of INTS12 binding (Additional file 1: Figure S10) prompted us to test for binding enrichment over the bodies of other gene classes. We tested protein coding, snRNA, small nucleolar RNA (snoRNA), microRNA, and long intergenic RNA (lincRNA) genes, and found that protein coding and snRNA genes show the highest enrichment for INTS12 binding (Fig. 6d). For protein-coding genes, peak binding is proximal to the TSS while for snRNA genes the binding is enriched downstream of the transcriptional end site (TES). Of note, the peak binding for lincRNA genes is near the TSS as for the protein coding genes. The enrichment near TES for snRNA genes is in agreement with INTS12 role as part of snRNA processing machinery which occurs simultaneously to the nascent transcription of 3’box elements [9]. In summary, the observed different patterns of binding over these protein coding and snRNA regions suggest distinct functional activities for INTS12 depending on the class of the genes it binds to.

### Association of INTS12 binding with specific regulatory elements

**Fig. 7:**
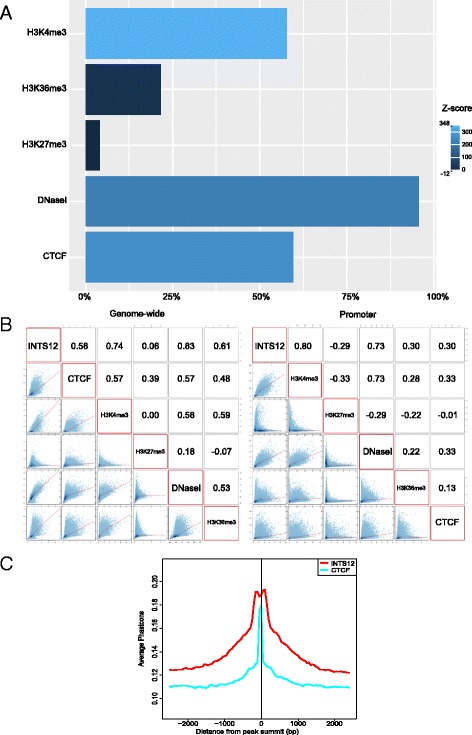
Summary of INTS12 binding with HBEC epigenetic regulatory elements. **a** Percent of total INTS12 binding sites overlapping with HBEC-specific regulatory elements. Data from the first donor is shown as a representative of the two donors tested. Colour indicates the Z-score of the distance between the observed overlap and the mean of distribution of random overlap permutations. Negative Z-score implies that the observed overlap is less than expected by chance. Higher Z-score implies larger distance to the mean of distribution in a randomization test. Within *P* < 0.05 the maximum Z-score in random permutation walk is 8, 6, 4, 7 and 3 for H3K4me3, H3K36me3, H3K27me3, DNaseI, and CTCF respectively. The features most prominently localizing with INTS12 are H3K4me3 (Z-score = 348) and DNaseI (Z-score = 223) both marking transcriptionally active regions as well as CTCF (Z-score = 264). **b** Cross-correlations of INTS12 and HBEC specific regulatory elements ChIPseq signals on a genome-wide scale and in the promoter regions (TSS ± 3000 bp). Numbers represent Pearson’s correlations between ChIPseq signals of respective reference datasets. **c** Evolutionary conservation of INTS12 binding sites in vertebrates. The figure is showing the average phastcons score derived from multiple sequence alignment of vertebrate genomes, across the binding sites of INTS12 (red) and CTCF protein (blue)

We next examined the localisation of INTS12’s binding in relation to specific regulatory elements identified in HBECs [45]. Because bioinformatic searches indicated INTS12’s PHD motif domain to be a candidate nucleosomal histone 3 tail binding protein, we tested the intersection of representative INTS12 binding with reference localizations of histone 3 lysine 4 trimethylation (H3K4me3), histone 3 lysine 36 trimethylation (H3K36me3), and histone 3 lysine 27 trimethylation (H3K27me3) modifications using per-chromosome randomization test [46] (Fig. 7a, Additional file 1: Figure S11). 58% of INTS12 binding co-localized with H3K4me3 (Z-score = 348), 21% with H3K36me3 (Z-score = 13), and 4% with H3K27me3 (Z-score = −12). Interestingly, 96% of INTS12 binding occurred in the vicinity of HBECs’ DNaseI accessible chromatin signature (Z-score = 223). INTS12 also overlapped with CTCF insulator protein at 60% of its binding sites (Z-score = 264).

In addition to testing the relationship between cross-binding of INTS12 and cell type variable mobile element sites we also examined the overall correlation of their respective ChIPseq signals on a genome-wide scale (Fig. 7b). In agreement with our initial observations, INTS12 signal most strongly correlated with accessible chromatin (*ρ* = 0.83) followed by H3K4me3 (*ρ* = 0.74). H3K36me3, CTCF and H3K27me3 had weaker correlations of 0.61, 0.58, and 0.06 respectively. Since gene-centric analysis revealed INTS12 binding to be enriched near TSS we also examined the correlation of ChIPseq signals at the promoters. In this analysis, the strongest correlation was observed between INTS12 and H3K4me3 (*ρ* = 0.80) outweighing the correlation between INTS12 and DNaseI (*ρ* = 0.73). Correlations with H3K36me3, CTCF and H3K27me3 were weak at the promoters being 0.3, 0.3, and −0.29 respectively.

Overall, based on these data, it is possible to say that INTS12 binding closely associates with the canonical marks of active transcription i.e. H3K4me3 and DNaseI. On a genome-wide scale INTS12 appears to be closely associated with DNaseI signature, while at the promoter regions INTS12 is more highly associated with H3K4me3 modification. The identified binding sites are likely to be biologically active as INTS12 peak regions (±500 bp in both directions from the peak summit) show stronger evolutionary conservation when compared with proximal neighbouring regions (Fig. 7c). INTS12 regions appeared more evolutionary conserved than CTCF sites, and interestingly, CTCF binding locations are much more narrowly conserved (±80 bp in both directions from the peak summit) than what is observed for INTS12. These representative observations from the first donor are recapitulated in the second donor and therefore our study provides supporting evidence of recruitment of INTS12 into transcriptionally active loci which may be modulated via its binding to histone 3 and recognition of H3K4me3 modification.

### Combination of ChIPseq and RNAseq reveals INTS12 regulome

**Fig. 8:**
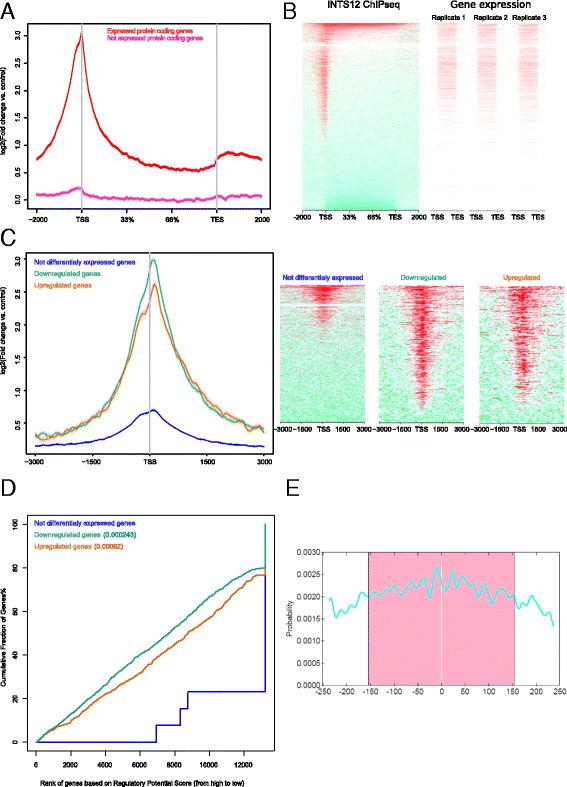
Combination of ChIPseq and RNAseq following INTS12 depletion defines INTS12 regulome and mode of action. **a** Log_2_ fold change of INTS12 ChIPseq binding signal versus input control across gene bodies of expressed and silenced genes in basal HBECs. **b** Comparison of INTS12 binding vs. corresponding gene expression in basal HBECs. Genes were ordered based on the level of INTS12 ChIPseq signal. The same sorted gene list was used to evaluate their transcription in basal un-transfected HBECs where red colour indicates higher expression derived from read counts on the corresponding gene bodies. **c** Average INTS12 binding profile for differentially expressed genes and genes with no evidence of differential expression following INTS12 depletion (left) as well as heatmap representation of this binding (right), with red indicating enrichment while green denoting lack of enrichment in ChIPseq sample versus input control. **d** Prediction of the activating and repressive function of INTS12. The cumulative fraction of genes is plotted against the regulatory potential, based on significance of representative D-siRNA A differential expression and ChIPseq evidence of binding near genes’ TSS. Regulatory potential is a product of the ranked potentials ($R P=\frac{rank\left({S}_{g\  binding}\right)}{n}*\frac{rank\left({S}_{g\  differential\  expression}\right)}{n}\ \Big)$ as judged by distance and number of INTS12 binding sites near gene’s TSS (*S*_*g binding*_ = ∑_*i* = 1_^*k*^ *e* ^− (0.5 + 4Δ*i*)^) and the potential as judged by significance of gene’s differential expression following INTS12 depletion (*S*_*g differential expression*_ = *Q* − *value*). INTS12 depletion was equally likely to induce or suppress gene expression in Kolmogorov-Smirnov test but >90% of downregulated genes had a higher regulatory potential than upregulated genes explaining the more robust effects observed on downregulated pathways. **e** Probability distribution of INTS12 binding enriched DNA motif TGAxTCA across the sites at which it is present. Position at zero represents peak summit and motif appears to be centrally enriched 158 bp in each direction from this summit. The site probability curve is broad (*P* = 2.7 × 10^−17^) indicating indirect or cooperative binding to the DNA

To provide insights into the dynamics of INTS12 regulation, we have overlaid ChIPseq and RNAseq datasets. As INTS12 showed the highest enrichment with DNaseI and H3K4me3 sites, both marking active transcription [47, 48], and poor correlation with H3K27me3, which marks silenced loci [47], we examined whether these observations agree with gene expression in basal HBECs. INTS12 had 8-fold higher enrichment of binding near the TSS of expressed genes (defined as having greater than zero fragments per kilobase per million reads (FPKM) in at least one biological replicate). On the other hand, INTS12 had only 1.2-fold enrichment of binding over silenced genes (defined as having zero FPKM in three biological replicates) (Fig. 8a). The magnitude of binding corresponded well with the degree of gene expression (Fig. 8b).

Next we aimed to identify the set of genes regulated by INTS12 (i.e. its regulome). Genes were divided into upregulated, downregulated and not differentially expressed following INTS12 knockdown. On average there was 6-fold, 8-fold and 1.6-fold enrichment of INTS12 binding above genome background near the TSS of upregulated, downregulated and not differentially expressed genes respectively (Fig. 8c). Thus of the total number of downregulated and upregulated genes 92 and 85% of genes show evidence of INTS12 binding near their promoters, while only 23% of genes that had no evidence of differential expression showed this localization. To provide validation for our findings we calculated the regulatory potential of INTS12 for each gene based on evidence of near promoter (TSS ± 1000 bp) binding as well as significance of differential expression following D-siRNAs depletions, and plotted the ranked list of genes based on their regulation versus cumulative fraction of genes having a given or higher regulatory potential score (Fig. 8d) [49]. Deregulated genes had significantly greater regulatory potential scores than static genes, with downregulated having lower P-value than upregulated. This shows that the genes with evidence of near promoter binding were contributing to the altered expression following INTS12 knockdown, with bias for supressed genes. Moreover, >90% of downregulated genes had higher regulatory potential than upregulated ones explaining more robust effects observed among the downregulated pathways.

### Motif enrichment and distribution uncovers INTS12 mode of action

We next sought to understand if INTS12 binds to DNA directly or in a cooperative fashion. Central motif enrichment analysis can identify whether the precipitated protein shows evidence of direct or cooperative DNA binding based on the probability distribution of enriched motif among its binding sites. Proteins with direct DNA binding, such as transcription factors, display binding sites clustering near the centres of the declared ChIPseq peaks [50] and we leveraged this method to test the most likely type of INTS12 binding. Using a differential analysis approach [51], we identified an enrichment for a motif among 20 and 12% of the total sites which occurred only among 6 and 5% of background genome sequences in the first and second donor respectively. The same signature was recapitulated by a separate non-differential algorithm [52]. The identified sequence was compared to currently known motifs [53] and was found to be identical to a motif previously found enriched among binding sites of activator protein 1 [54], activating transcription factor 3, nuclear basic leucine zipper, jun dimerization protein 2 [55, 56], nuclear factor erythroid 2 as well as Fos-related antigen 2 [45]. Although this motif appears to be centrally distributed, the site probability is relatively broad (Fig. 8e) suggesting that much of the binding via the identified motif occurs in cooperation with other molecules. We conclude that among the sites where the identified enriched motif occurs, INTS12 does not have the characteristics of a transcription factor and is more likely to act as a co-factor in concert with other molecules.

## Discussion

Our study provides key insights into the molecular and cellular functions as well as the regulatory properties of *INTS12*, a candidate lung function gene. Through lung eQTL approach we present evidence that SNPs associated with pulmonary parameters also correlate with *INTS12* expression, which is not the case for the neighbouring *GSTCD* gene. eQTL analyses have suggested that it is the altered expression of INTS12 as a more likely driver of the genetic association for lung function, but this has been based on the data obtained from non-lung tissue [7]. Due to the diversity of tissue gene expression, it is of pivotal importance to use phenotype-relevant datasets [20]. A previous investigation of a lung microarray dataset [55, 56] failed to detect a significant eQTL effect on INTS12. This may have been due to the technical heterogeneity of hybridization-based array assays [57]. Using a more sensitive lung RNAseq dataset, in our eQTL analysis we were able to detect the effect of lung function SNPs on INTS12 expression. It has become largely accepted that INTScom exerts its effects via snRNA processing pathway, however we find that in HBECs, among U1, U2, U4 and U5 species, only U2 3’-end formation is affected following INTS12 knockdown.

### The contribution of INTS12 to human snRNA processing

INTS12 is a member of the INTScom which itself has been shown to be implicated in numerous molecular and cellular processes. It remains unclear whether all INTScom subunits are required for some of these processes, especially that there is variability in the relative contributions of various complex members to snRNA processing [9–12], maintenance of perinuclear dynein [13] and ciliogenesis [17]. What remains to be elucidated is how INTScom perturbations yield specific yet diverse phenotypes.

It has been suggested that the primary mechanism behind that is the alteration of snRNA 3’-end formation affecting the splicing of mRNAs belonging to genes of particular functional groups explaining the specific phenotypic effects [13, 15, 16]. For instance, it has been argued that the induced downregulation of INTS5, INTS9, and INTS11 in zebrafish causes impaired haematopoiesis due to aberrant splicing of smad1 and smad5 via a dominant negative form of these transcripts [16]. However, given the facts that INTS11 depletion results in a loss of perinuclear dynein whilst there was no enrichment for misprocessed transcripts encoding dynein-dynactin subunits, adaptor molecules or dynein-binding cassettes in HeLa cells [13] and our own observation of minor effect of INTS12 knockdown on snRNA processing concurrent with misbalanced protein synthesis, this hypothesis seems unlikely in a human model. This is further supported by a literature review of studies that compared the contribution of various INTScom members to snRNA processing, showing INTS12 to have a fairly small role in comparison to other members of the complex (Additional file 2: Table S6). Moreover, in the overrepresentation analysis of genes with evidence of D-siRNAs-reproducible altered splicing we found a poor enrichment of only immune response pathways (e.g. class I MHC mediated antigen processing and presentation; FDR < 0.05) but none of genes with altered splicing were part of any of the identified protein synthesis pathways (data not shown). Therefore, partly based on our observation of poor correlation of INTS12 levels with the rest of the complex in our datasets, alternatively we propose INTScom subunits to have different activities despite their physical association in the same complex and with POLII. Consequently, the prediction from this model would be that individual INTScom members are pleiotropic [58] and have distinct functions which may explain the plethora of phenotypes observed following various perturbations of INTScom.

### Novel functional roles for INTS12

We have relied on a hypothesis-free approach [20] in order to generate new functional hypotheses about INTS12 function. Following its knockdown with two D-siRNAs, we observed marked downregulation of pathways critical in protein synthesis including tRNA synthetases, unfolded protein response and PERK pathways. To further investigate the importance of this, we undertook additional experiments which showed that suppression of INTS12 reduces protein synthesis and proliferative capacity. Thus the identified molecular signature affected a relevant phenotype, uncovering a new function for this gene by demonstrating its role in regulating cellular translation. We have also detected upregulation of collagen formation and extracellular matrix deposition, but the effects upon the upregulated pathways were less robust.

We then performed INTS12 ChIPseq to delve deeper into the mechanism behind the identified gene expression changes. The top three fixed genomic features associated with INTS12 binding were introns, intergenic regions and promoters. A gene-centric analysis shows a distinct localization near the TSS and TES for protein coding and snRNA genes respectively. Moreover, INTS12 interaction was enriched for canonical epigenetic marks of transcription. The combined RNAseq and ChIPseq analyses revealed preferential INTS12 binding to the expressed rather than silenced genes and defined its regulome which includes genes belonging to the aforementioned pathways.

### INTS12 in evolution and development

It has been hypothesised that the mechanisms involved in the early human lung development may alter lung function and predispose to COPD later in life [59]. Although a subset of lung function associated genes show evidence of differential expression between various stages of embryonic pulmonary tissue formation [20], there is still an incomplete understanding of the molecular mechanisms behind normal respiratory system development and how the alterations therein contribute to disease pathophysiology. Given that there is no homologous *INTS12* in unsegmented *C. elegans* or unicellular *S. cerevisiae*, its strong conservation and lethal effect of knockout in *M. musculus*, this gene may have been important for the evolution of complex metazoan tissue differentiation and specialization. This is also supported by our observation of INTS12 binding association with canonical epigenetic marks of transcription which are known to be reset during mammalian organogenesis [60]. It therefore seems plausible that *INTS12* regulates lung development or repair via a developmental pathway.

### INTS12 knockdown induced gene dysregulation of relevance to pulmonary physiology

INTS12 knockdown for 48 h and 120 h resulted in reproducible dysregulation of core subset of genes important in airway biology, such as *SERPINA1*, *TGFβI*, *IL6*, and *IL8*. Of particular interest is *LEP* which had 4.51 and 29.16-fold upregulation relative to control in D-siRNA A condition at 48 h and 120 h time points respectively. *LEP* associates with the same lung function parameter as *INTS12* (6, 33) albeit weaker than what was reported for 4q24 locus. Crucially LEP levels negatively correlate with lung function [34]. It is possible that reduced levels of INTS12 in specific allele carriers are responsible for elevated expression of *LEP* which may in turn account for reduced lung function. These causal hypotheses provide biological understanding of the genetic association signal for pulmonary function but require further exploration.

## Conclusion

We conclude that INTS12 is a pleiotropic gene with at least two different functions depending on the class of genes where its binding occurs. In agreement with the canonical function, over snRNA genes INTS12 is likely to contribute to their 3’-end formation. However, in contrast to what was reported in *Drosophila*, INTS12 requirement for snRNA processing is moderate in human cells highlighting differences between these two species. Our data identify a significant and previously unrecognized role for INTS12 in protein synthesis control. A novel INTS12 regulome was uncovered and implies a regulation of protein coding genes belonging to the translational pathways.

## Methods

### Expression quantitative trait locus analysis

To functionally elucidate the GWAS signal for lung function at 4q24 locus, a lung specific and RNAseq based *cis*-eQTL dataset [21] was used. The nominal *P*-values from testing the association between SNP variation and gene expression were obtained and corrected in R using Benjamin-Hochberg FDR correction [61]. The FDR values from eQTL analyses of 4q24 SNPs were compared to the significance of association with lung function parameter forced expiratory volume in a first second from the Repapi et al. study [3].

### RNA interference

The purpose of RNAi experiments was to deplete cells of INTS12 in order to (a) study it in the context of existing body of knowledge to translate canonical activity into a human model, (b) predict novel functions based on transcriptomic profiling, (c) and to test them experientially. To help distinguish between true and off-target effects, gene knockdown was performed using two independent D-siRNAs. Experiments included un-transfected and scrambled D-siRNA transfected controls. RNAseq profiling was performed 48 h and 120 h after the initiation of RNAi, to compare the transcriptomic responses at these two time points. RNAseq pathway analyses, functional and validation experiments were assessed using 120 h long interference. INTS12 depletion was performed in discovery and independent validation donor HBECs using a minimum of three biological replicates.

### RNAseq

RNA extraction from knockdown and control conditions, cDNA library preparation and next generation sequencing are described in Additional file 3: Supplemental Methods.

### Quantitative PCR

U1, U2, U4 and U5 snRNA processing was assessed by measuring qPCR-estimated relative levels of their respective misprocessed transcripts. *MARS*, *GARS*, *ASNS*, and *ATF4* expression was also measured by qPCR for technical validation on the cDNA samples derived from RNA sequenced total RNA samples, and for biological validation in different donor cells using the same experimental design. Details of cDNA synthesis and qPCR assays are described in Additional file 3: Supplemental Methods.

### RNAseq and pathway data analyses

Detailed description of RNAseq and pathway analyses is in the Additional file 3: Supplemental Methods.

### ChIPseq and ChIP-PCR data analyses

ChIPseq and ChIP-PCR experimental procedures as well as detailed description of ChIPseq data analyses are described in Additional file 3: Supplemental Methods.

### Functional assays

Details about protein synthesis measurement and analyses are in the Supplemental Methods. Proliferative capacity was assessed by cell counts and details are described in Additional file 3: Supplemental Methods.

### Immunofluorescence

Antibodies, immunofluorescence methods, and detection are described in Additional file 3: Supplemental Methods.

### Statistics

Data were grouped from multiple experiments and are expressed as average ± standard error of mean. Statistical significance was assessed by ordinary one-way ANOVA followed by Fisher’s Least Significant Difference test. Results were considered significant when *P* < 0.05. For high throughput analyses the nominal P-values were corrected for multiple comparisons using FDR correction. The significance of dN/dS ratios in the selection test, was obtained via the Single-Likelihood Ancestor Counting algorithm [62].

## Additional files

Additional file 1: Figure S1–Figure S12. Contains all supplemental figure data. Each figure has its legend. (PDF 3851 kb)

Additional file 2: Table S1–Table S6. Contains all supplemental table data. Each table has its legend. (PDF 166 kb)

Additional file 3: Contains a more detailed supplemental information in relation to the methods: Cell Culture, RNAi, RNAseq, qPCR, RNAseq and Pathway Data Analysis [63], Protein synthesis by 35S-Methionine incorporation assay [64], Assessment of proliferative capacity by cell counts, ChIPseq, ChIP-PCR, ChIPseq Data Analysis [65–68], ENCODE data retrieval and analysis [69], Immunofluorescence [70]. (PDF 89 kb)

## Abbreviations

*ASNS*: Asparagine Synthetase
*ATF4*: Activating transcription factor 4
ChIPseq: Chromatin immunoprecipitation and sequencing
*cis*-eQTL: Nearby expression quantitative trait locus
COPD: Chronic Obstructive Pulmonary Disease
CPM: Counts per methionine
CTCF: CCCTC-binding factor
D-siRNA: Dicer substrate small interfering RNA
eQTL: Expression quantitative trait locus
*GARS*: Glycyl-tRNA synthetase
GSEA: Gene set enrichment analysis
*GSTCD*: Glutathione S-transferase, C-terminal Domain Containing
GWAS: Genome-wide association studies
H3K27me3: Histone 3 lysine 27 trimethylation
H3K36me3: Histone 3 lysine 36 trimethylation
H3K4me3: Histone 3 lysine 4 trimethylation
HBEC: Human bronchial epithelial cell
*IL1R1*: Interleukin 1 receptor 1
*INTS12*: Integrator Complex subunit 12
INTScom: Integrator Complex
*LEP*: Leptin
lincRNA: Long intergenic RNA
*MARS*: Methionyl-tRNA synthetase
PHD: Plant homeodomain
POLII: RNA polymerase II
qPCR: Quantitative polymerase chain reaction
RNAi: RNA interference
RNAseq: RNA sequencing
*SERPINA1*: α1-antitrypsin
snoRNA: Small nucleolar RNA
SNP: Single nucleotide polymorphism
snRNA: Small nuclear RNA
TES: Transactional end site
*TGFβI*: Transforming growth factor β 1
trans-eQTL: Distant eQTL
TSS: Transcriptional start site
