# Supplementary material for: Lung function associated gene Integrator Complex subunit 12 regulates protein synthesis pathways
Source: BMC Genomics. 2017 Mar 23;18:248. doi: 10.1186/s12864-017-3628-3 (PMC5364626; doi:10.1186/s12864-017-3628-3)

Supplemental Figures

Lung function associated gene Integrator Complex subunit 12 regulates protein synthesis pathways

Kheirallah et al.

Figure S1: Qualitative comparison of biological replicates of INTS12 protein knockdown.

Representative images from the first experiment are shown in the first column and are compared to the images from the independent experiment shown in the second column. Based on these observations it is possible to say that D-siRNA treatment resulted in INTS12 protein depletion and indicates the specificity of used antibody as there is a notable decrease of staining among cells in which RNAi was initiated. In agreement with previous reports, INTS12 appears to have a nuclear sub-cellular localization.

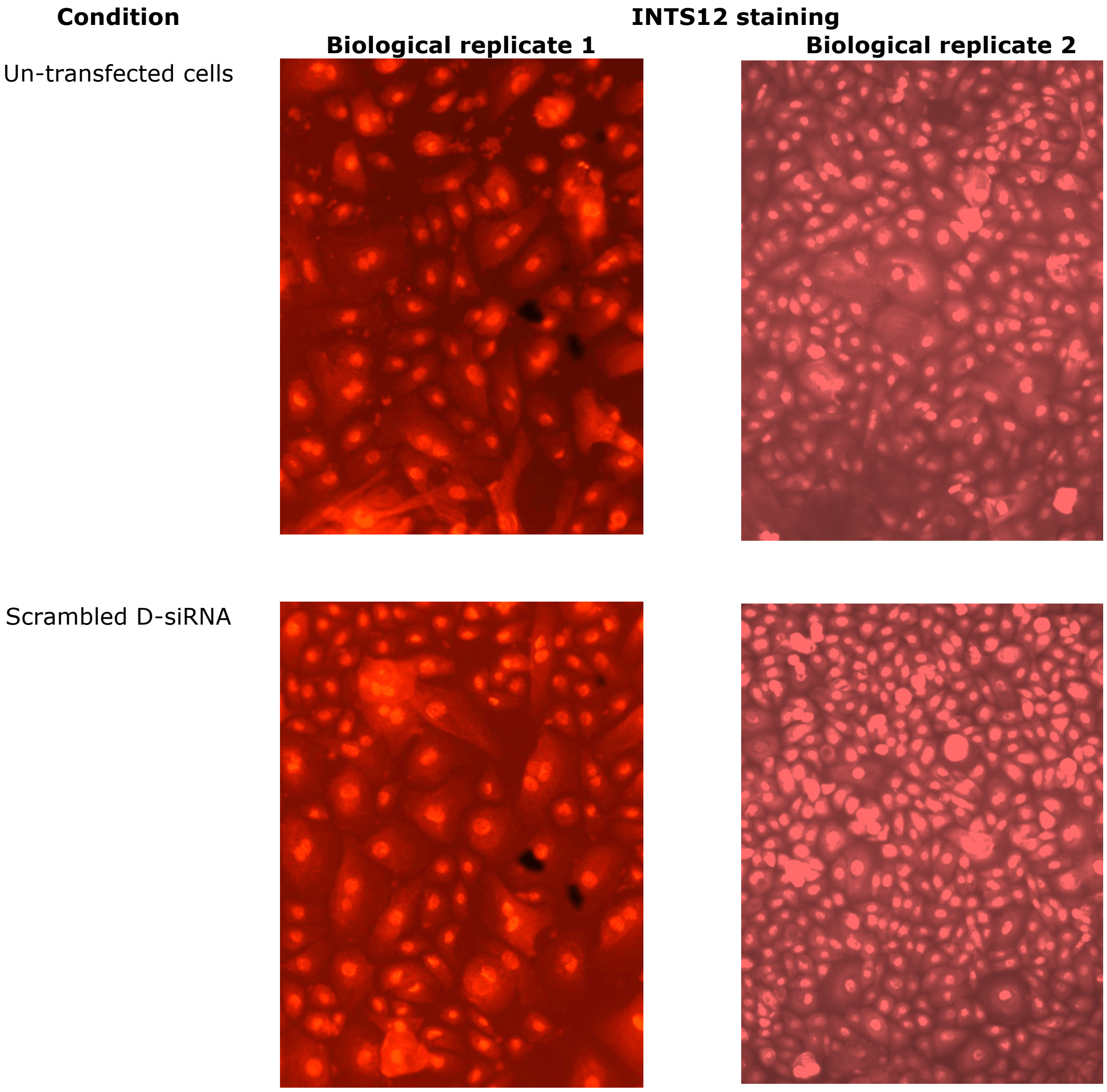

D-siRNA INTS12 knockdown

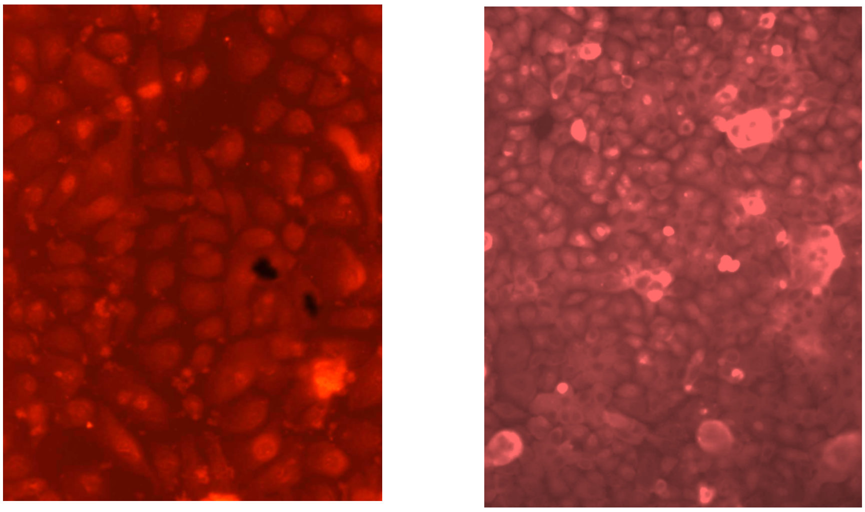

Isotype control

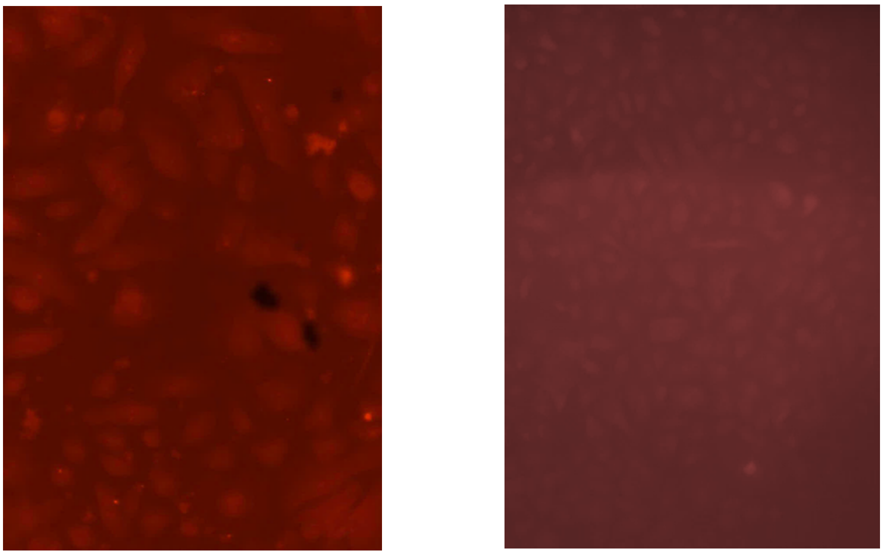

Figure S2: qPCR expression profiling of LEP expression in additional donor cells.

LEP is significantly upregulated in validation donor HBECs depleted of INTS12. Statistical tests were performed comparing to scrambled D-siRNA control: *P<0.05, ****P<0.0001. Individual ΔΔCt gene expressions are *GAPDH* normalized and relative to the mean of the scrambled D-siRNA condition. No significant difference was observed between un-transfected and scrambled D-siRNA transfected cells.

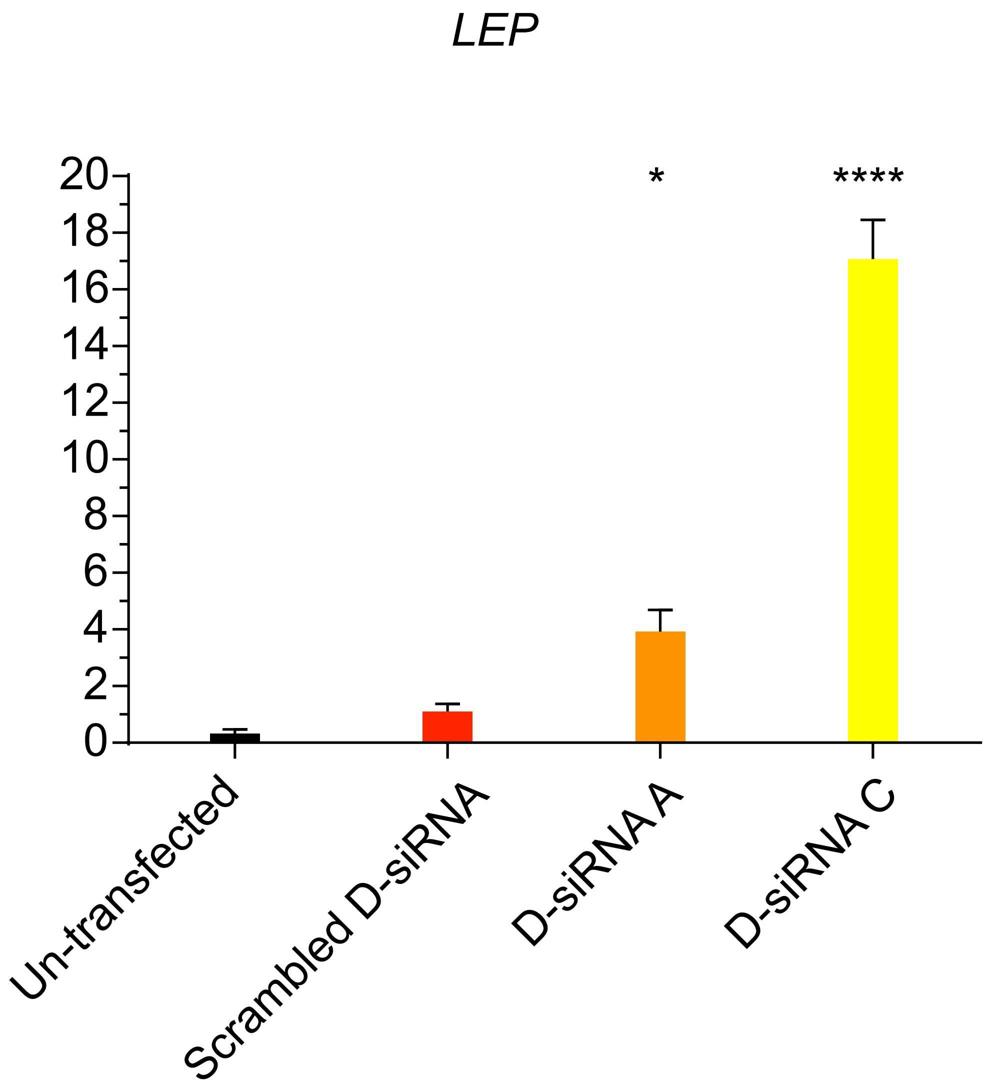

Figure S3: Box plots representing $\log_{10}$ of RNAseq FPKM expression values of genes belonging to the top dysregulated pathways.

Stars indicate the GSEA-derived significance of pathway dysregulation.

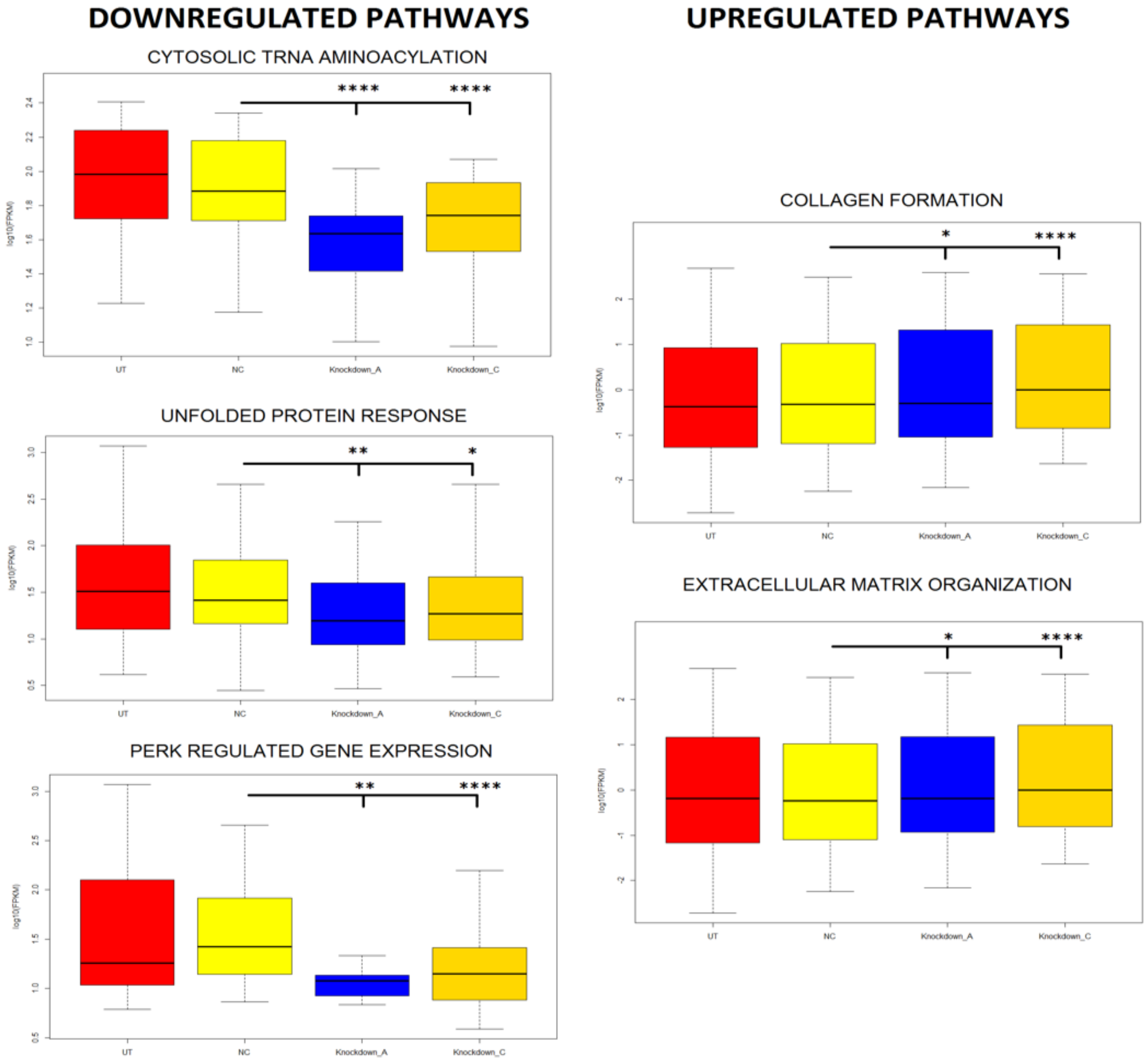

Figure S4: Enrichment plots of pathways upregulated by INTS12 knockdown.

Enrichment plots of reproducibly upregulated pathways in D-siRNA A and C analyses are shown with indicated statistical significance and normalized enrichment scores of their respective upregulations. The FDR and normalized enrichment score values were rounded up to one and three significant figures respectively.

**D-siRNA A analysis**	**D-siRNA C analysis**

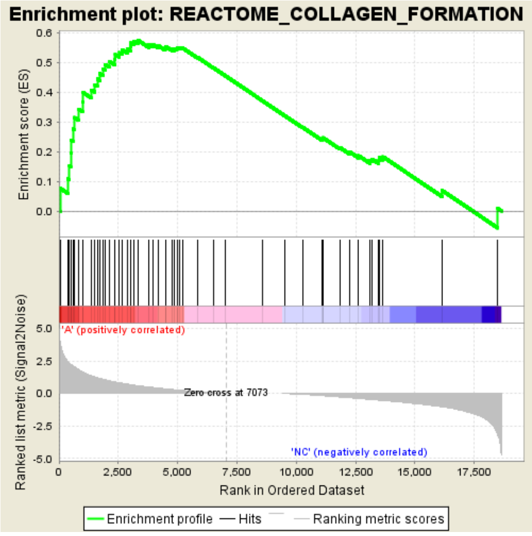

FDR = 0.01

Normalized enrichment score = 1.87

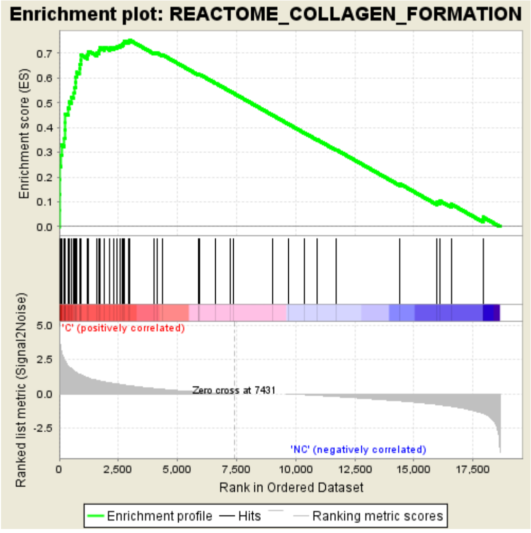

FDR < 0.00001

Normalized enrichment score = 2.42

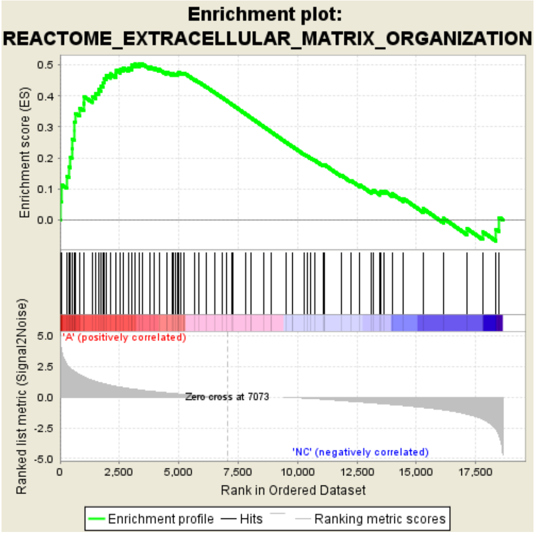

FDR = 0.03

Normalized enrichment score = 1.78

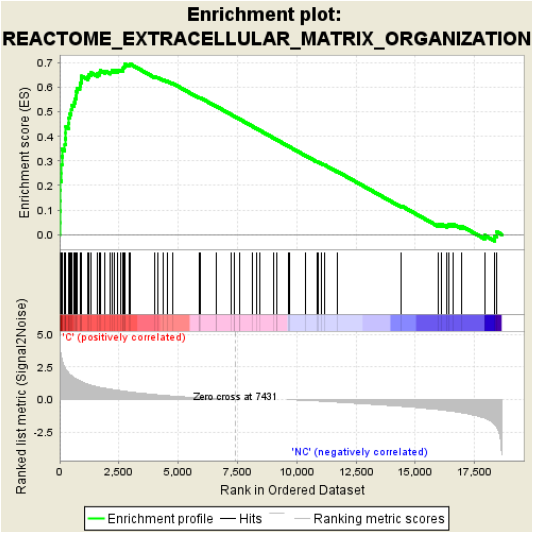

FDR < 0.00001

Normalized enrichment score = 2.40

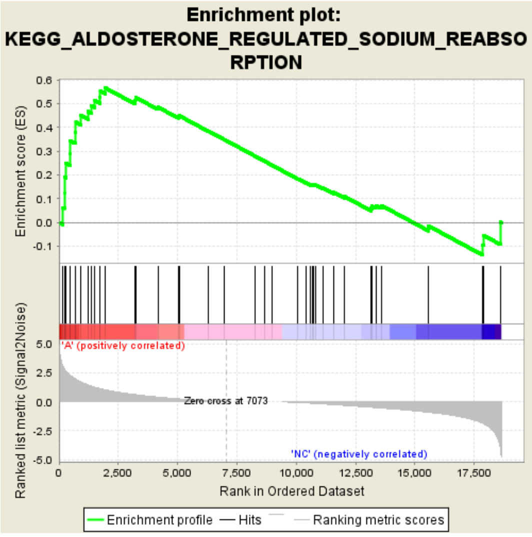

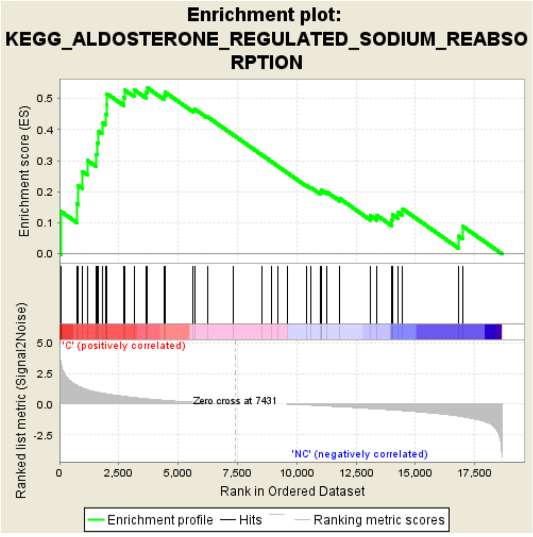

FDR = 0.04

Normalized enrichment score = 1.74

FDR = 0.05

Normalized enrichment score = 1.64

Figure S5: Gene expression heatmaps of genes belonging to reproducibly upregulated pathways.

Green and red colours on the Z-scale indicate lower and higher expression respectively. Samples were clustered by unsupervised hierarchical clustering and resulted in clustering of three biological replicate samples of each of the four conditions: un-transfected cells (UT), cells transfected with scrambled D-siRNA control (NC), cells transfected with anti-INTS12 D-siRNA A (A) and cells transfected with anti-INTS12 D-siRNA C (C).

Extracellular matrix organization (REACTOME)

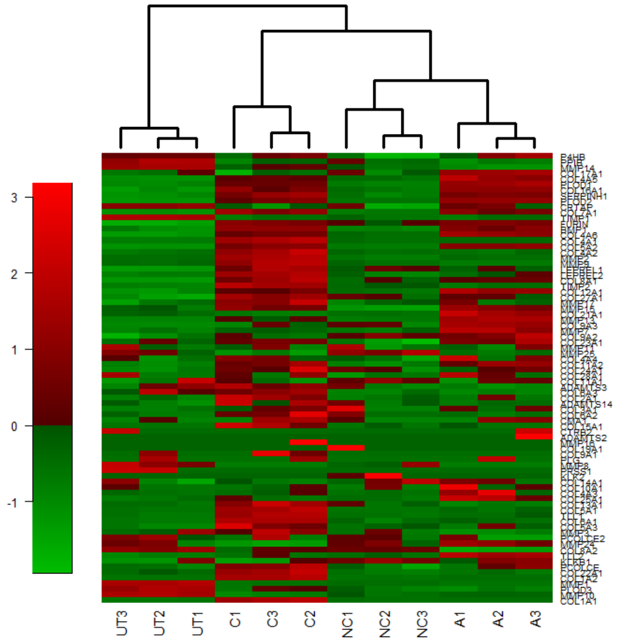

Collagen formation (REACTOME)

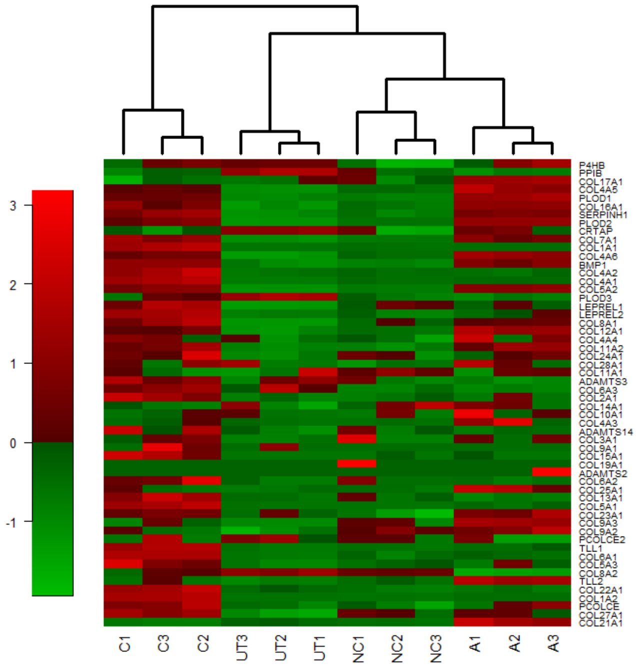

Aldosterone regulated sodium re-absorption (KEGG)

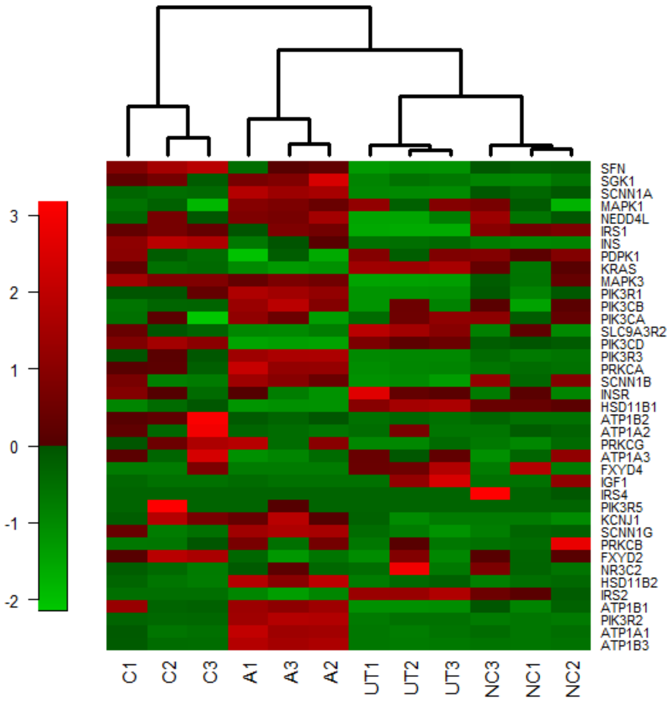

Figure S6: Enrichment plots of pathways downregulated by INTS12 knockdown.

Enrichment plots of reproducibly downregulated pathways in D-siRNA A and C analyses, except cytosolic tRNA aminoacetylation (REACTOME) and PERK regulated gene expression (REACTOME), are shown with indicated statistical significance and normalized enrichment score of their respective downregulations. The FDR and normalized enrichment score values were rounded up to one and three significant figures respectively.

D-siRNA A analysis

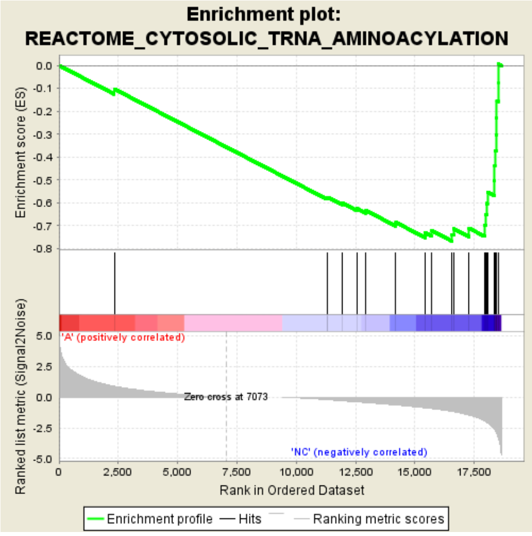

FDR = 0.0004

Normalized enrichment score = -2.05

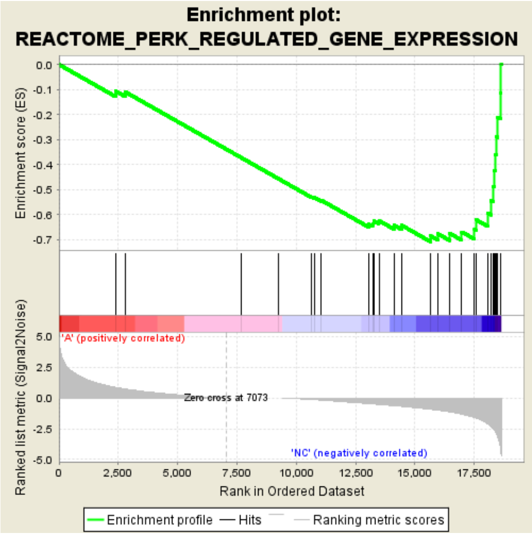

FDR = 0.002

Normalized enrichment score = -1.95

D-siRNA C analysis

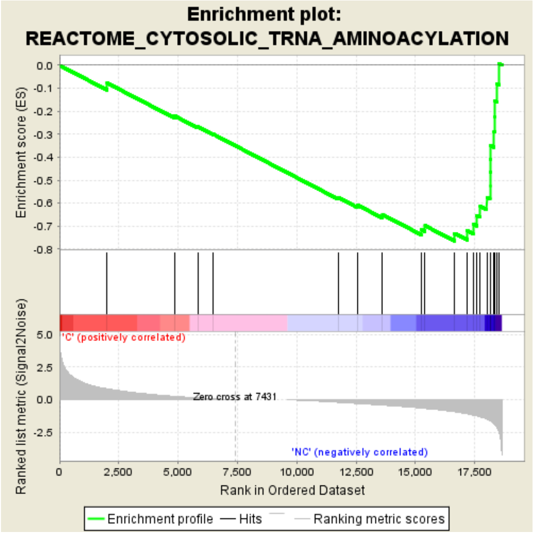

FDR = 0.00009

Normalized enrichment score = -2.10

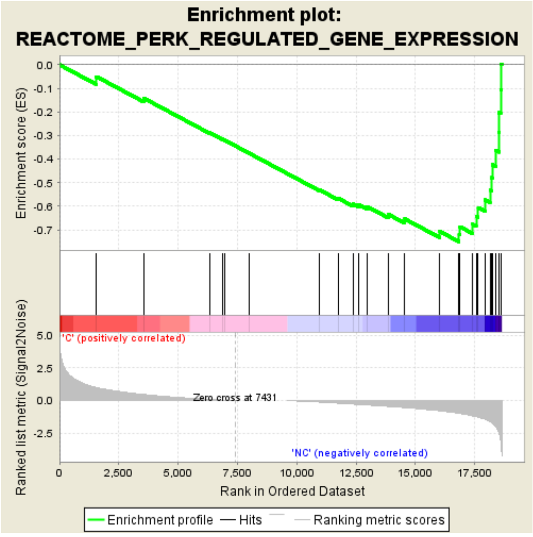

FDR = 0.00006

Normalized enrichment score = -2.12

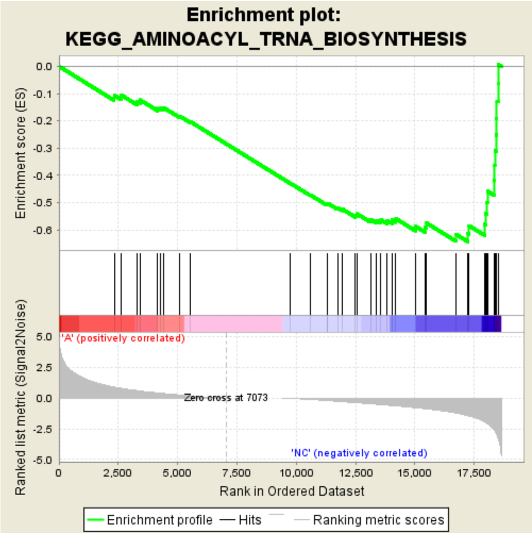

FDR = 0.003

Normalized enrichment score = -1.90

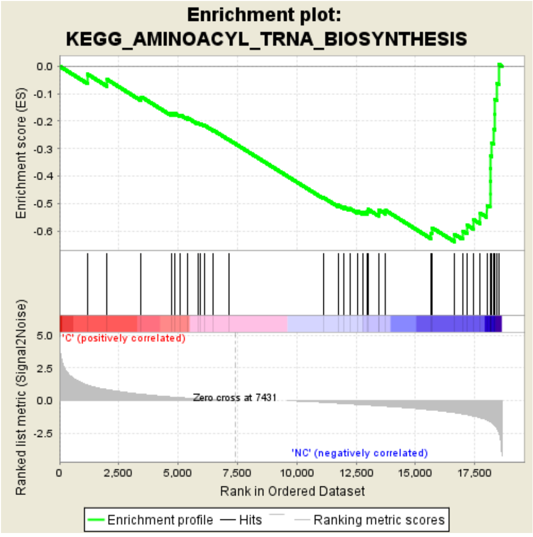

FDR = 0.004

Normalized enrichment score = -1.92

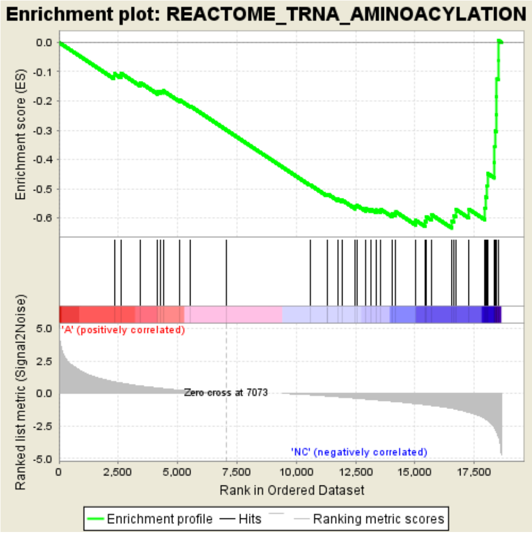

FDR = 0.004

Normalized enrichment score = -1.89

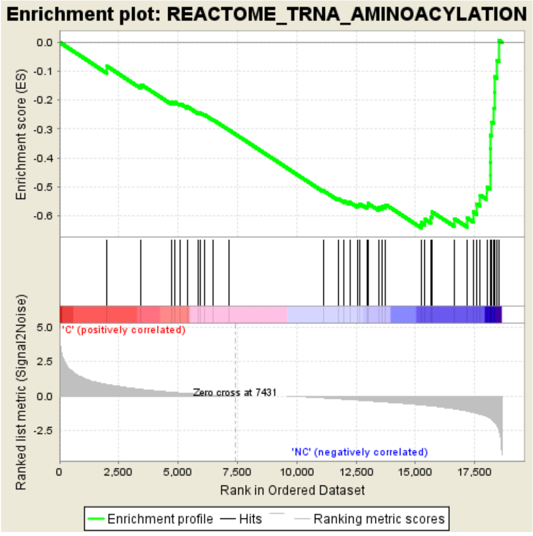

FDR = 0.003

Normalized enrichment score = -1.94

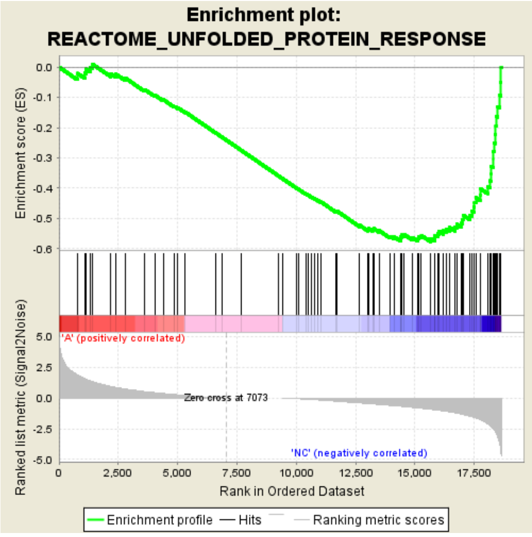

FDR = 0.003

Normalized enrichment score = -1.91

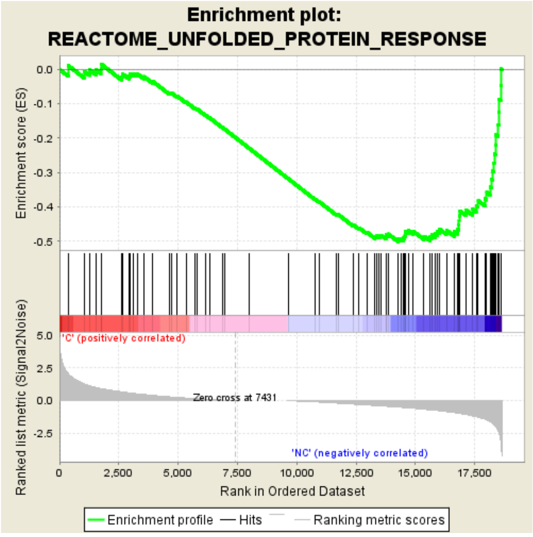

FDR = 0.03

Normalized enrichment score = -1.72

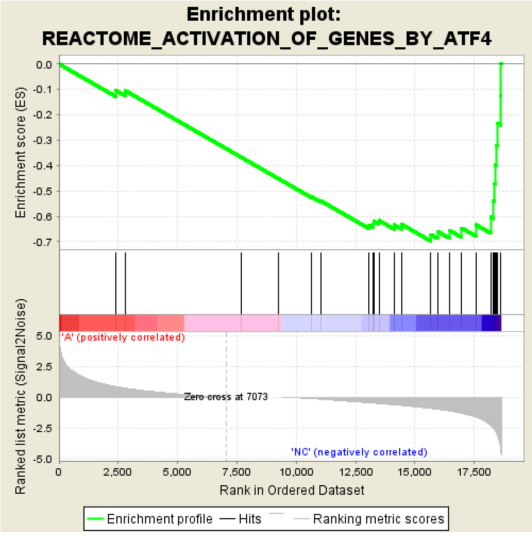

FDR = 0.006

Normalized enrichment score = -1.86

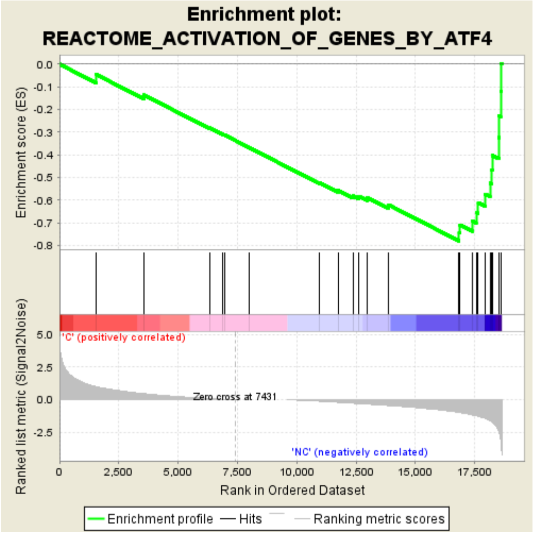

FDR = 0.0001

Normalized enrichment score = -2.10

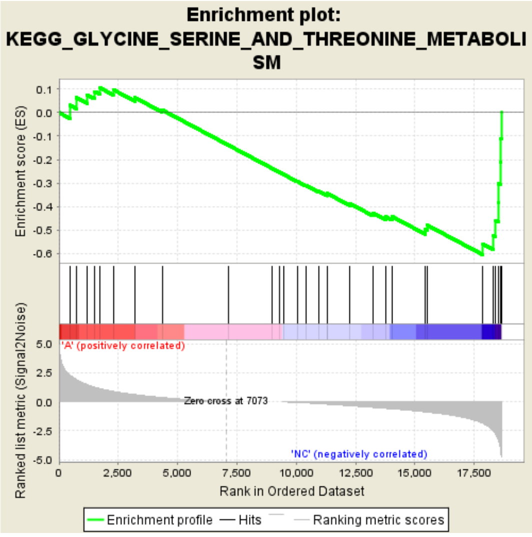

FDR = 0.03

Normalized enrichment score = -1.72

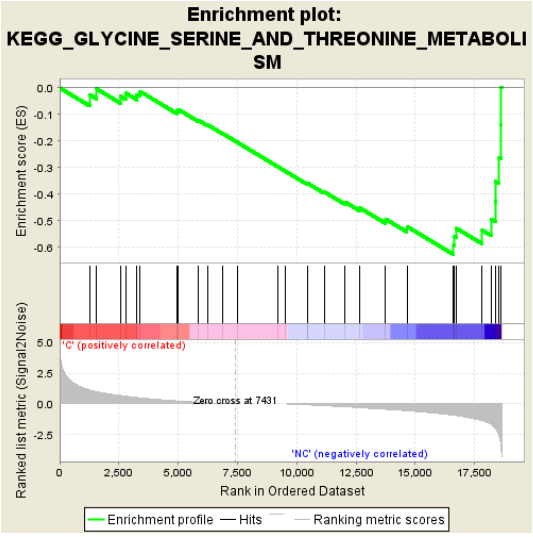

FDR = 0.02

Normalized enrichment score = -1.79

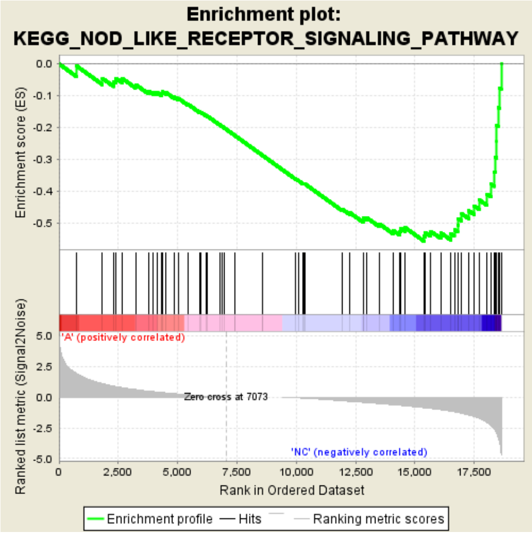

FDR = 0.01

Normalized enrichment score = -1.80

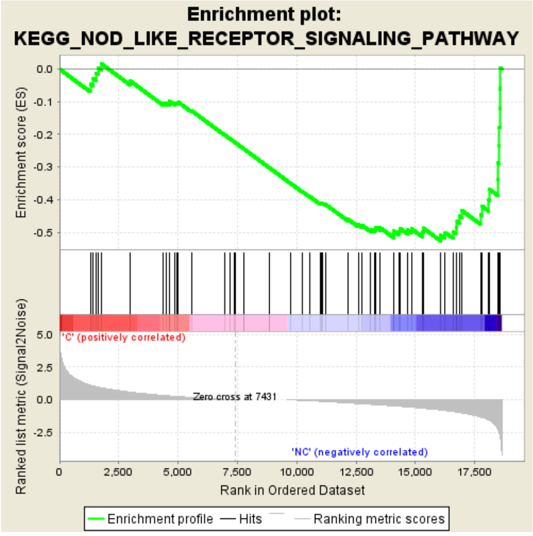

FDR = 0.03

Normalized enrichment score = -1.74

Figure S7 Gene expression heatmaps of genes belonging to reproducibly downregulated pathways.

Green and red colours on the Z-scale indicate lower and higher expression respectively. Samples were clustered by unsupervised hierarchical clustering and resulted in clustering of three biological replicate samples of each of the four conditions: un-transfected cells (UT), cells transfected with scrambled D-siRNA control (NC), cells transfected with anti-*INTS12* D-siRNA A (A) and cells transfected with anti-*INTS12* D-siRNA C (C).

Unfolded protein response (REACTOME)

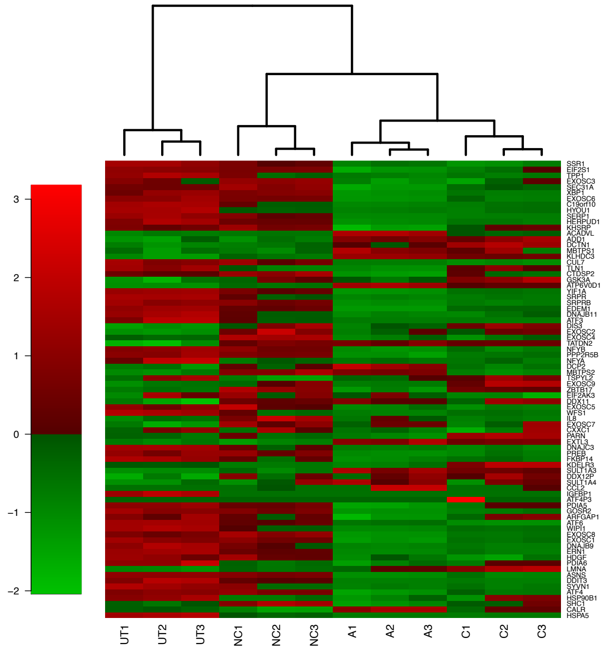

Activation of genes by ATF4 (REACTOME)

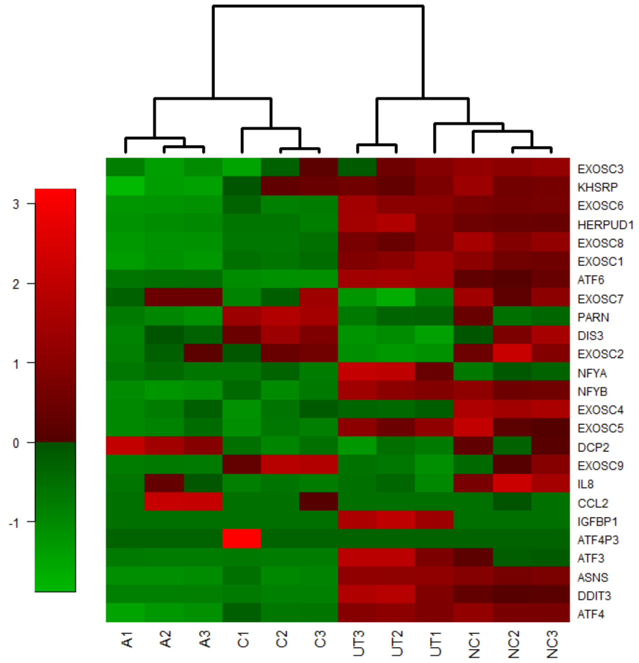

Glycine, serine and threonine metabolism (KEGG)

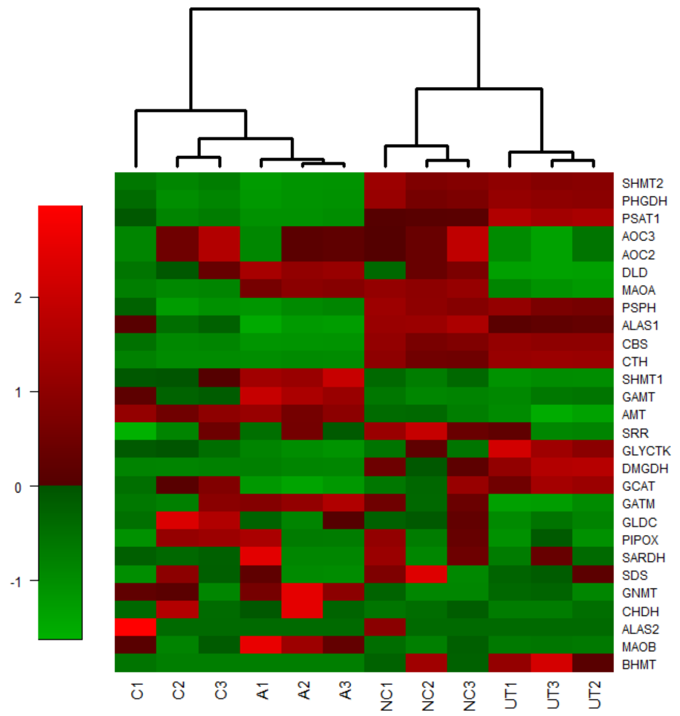

Aminoacyl tRNA biosynthesis (KEGG)

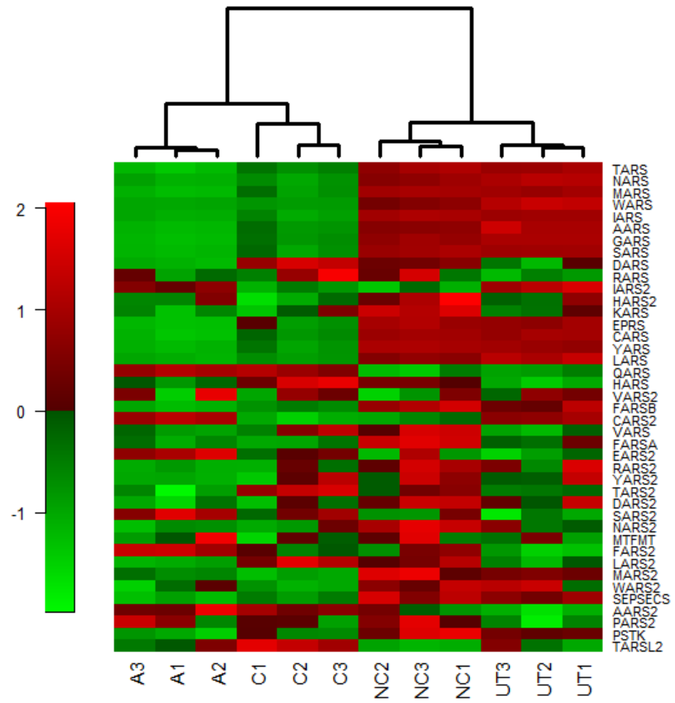

NOD like receptor signalling (KEGG)

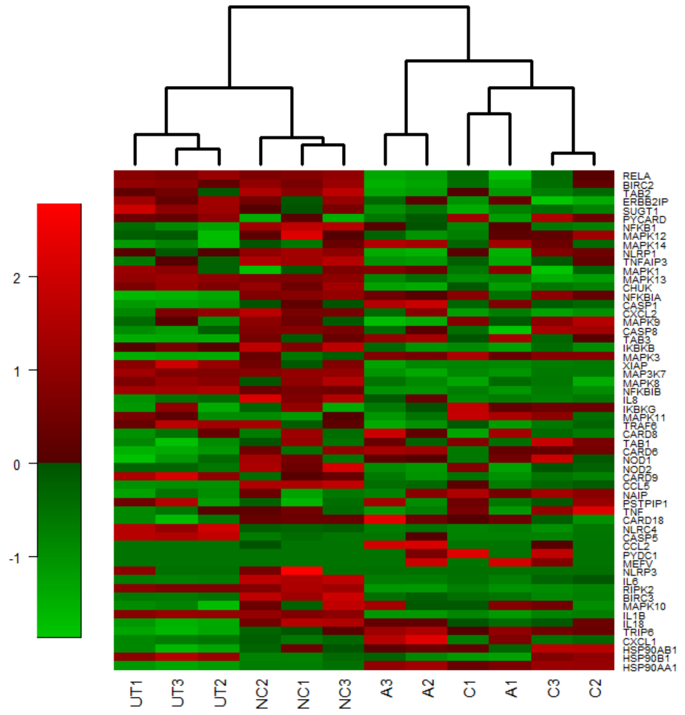

Figure S8: Biological reproducibility of INTS12 knockdown.

Correlation of ChIPseq signals in donor replicates of active regions defined by the start coordinate of the most upstream interval and the end coordinate of the most downstream interval (a union of donor 1 and donor 2 intervals) revealed a Pearson's correlation of 0.85 (P<0.0001).

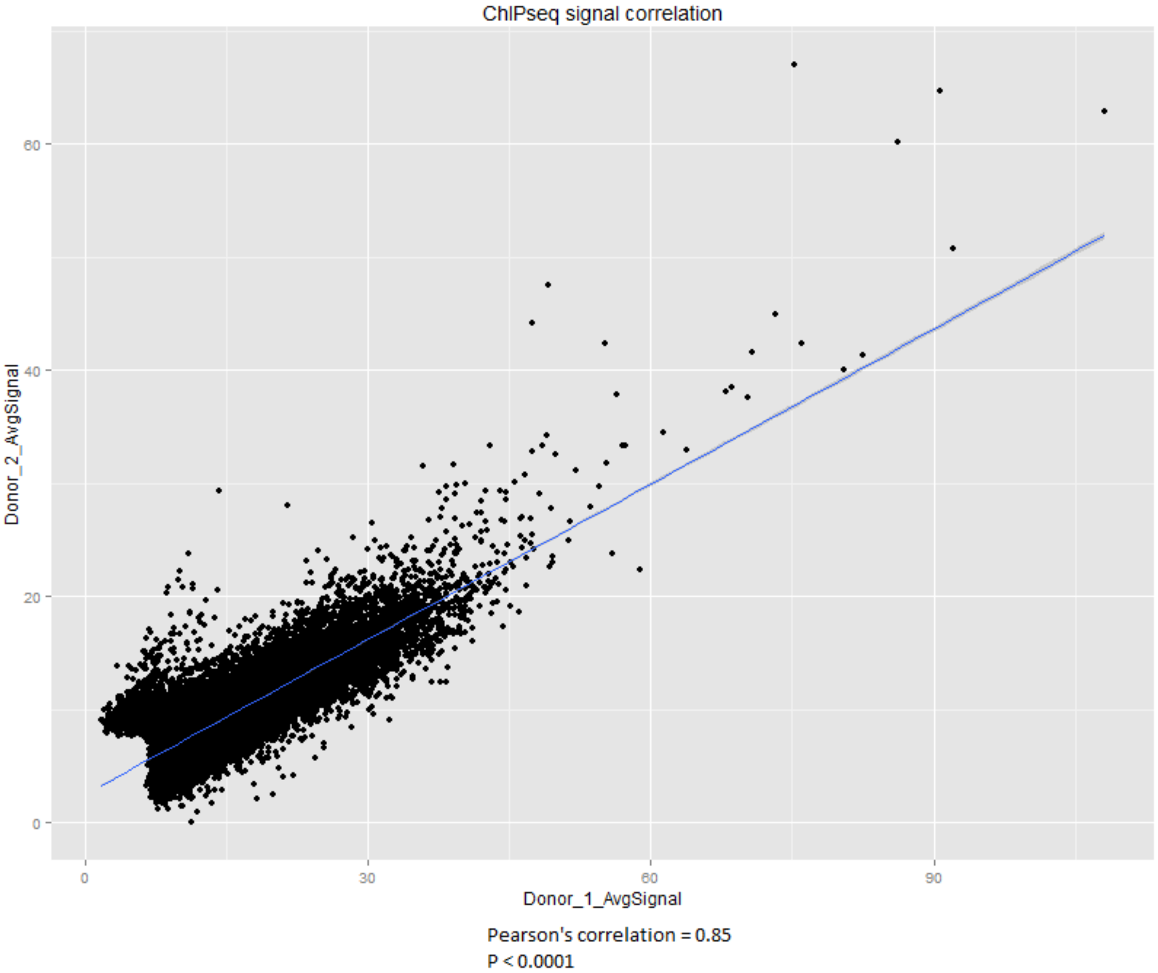

Figure S9: ChIP-PCR validation of ChIPseq findings.

Three ChIPseq positive binding sites (*POR*, *ACTB*, *NBPF1*) shown in green boxes and one negative binding site (*Untr12*) shown in blue box were selected for ChIP-PCR testing to determine the number of binding events detected per thousand donor 1 (D1) and donor 2 (D2) cells. ChIP-PCR results corresponded well with ChIPseq data as seen on the genome browser.

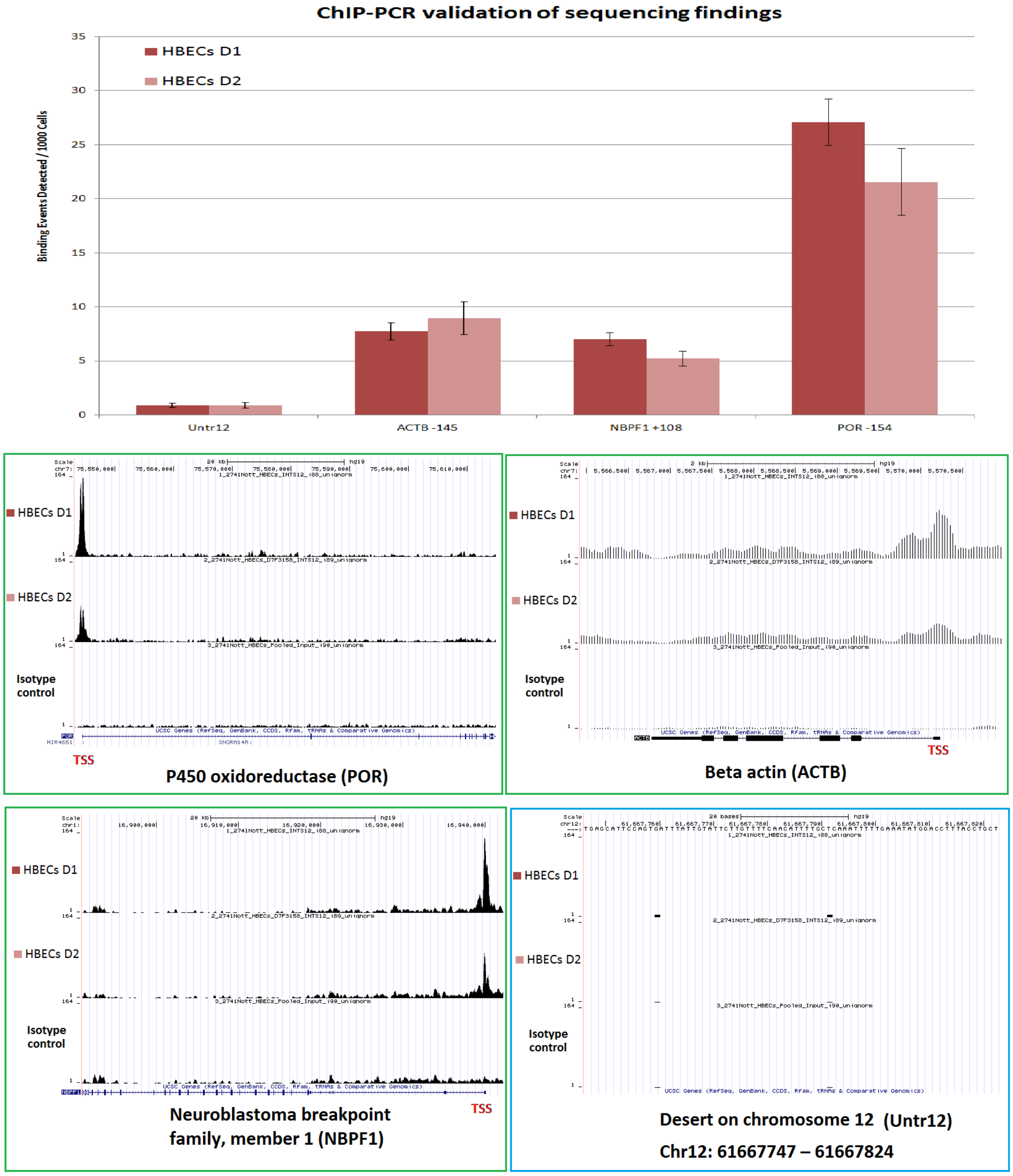

Figure S10: INTS12 ChIPseq peaks over the human genome in donor 1 and donor 2 cells.

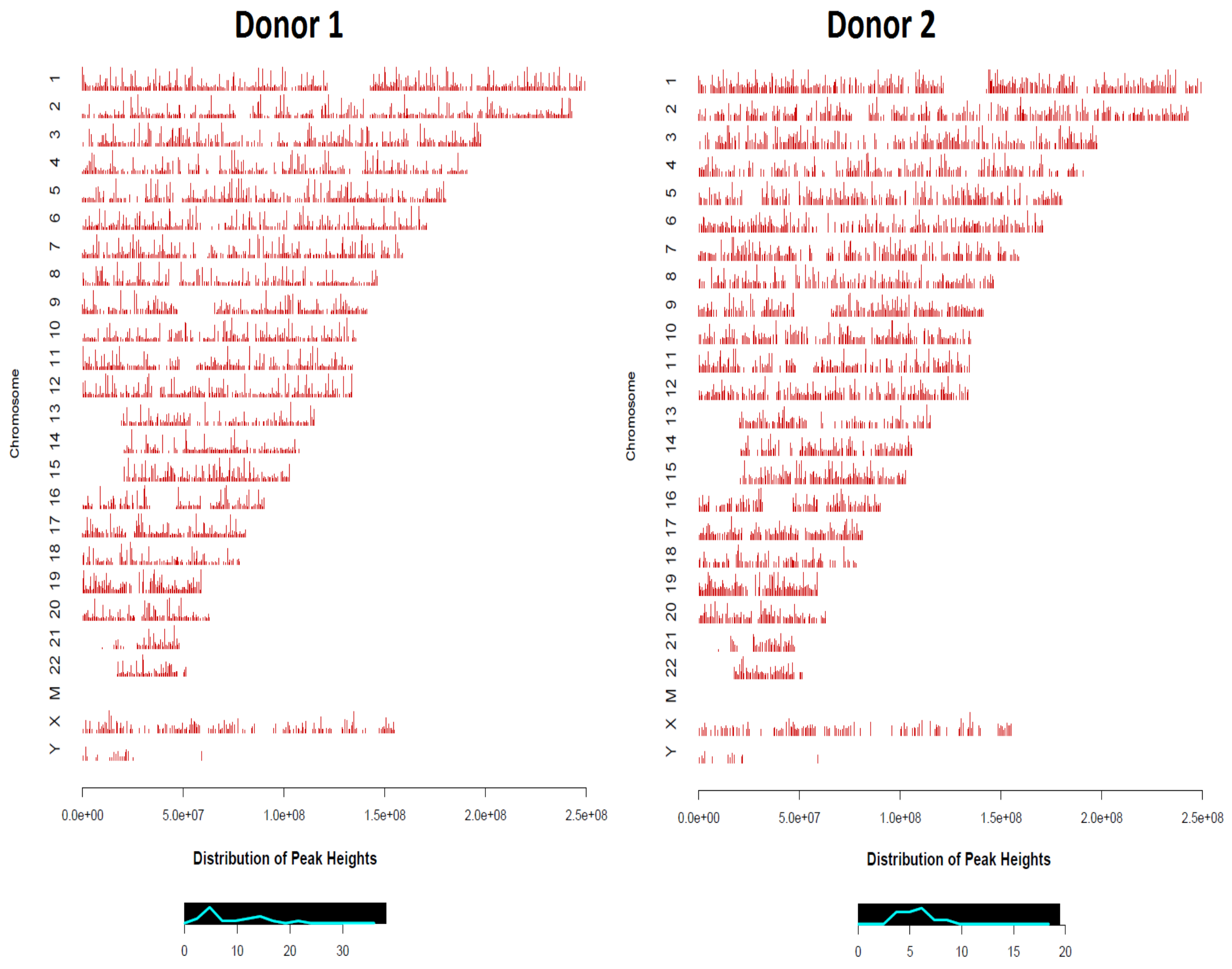

Figure S11: The distance between the average of distribution of intersection in random shuffling of target sites and the observed number of overlaps with INTS12 binding.

Random walk represents the frequency distribution of overlaps between INTS12 and test regions generated by shuffling H3K4me3, H3K36me3, H3K27me3, DNaseI and CTCF sites in thousand times permutation test. The larger the Z-score distance between the observed and permuted distribution of intersection, the less likely it is to have occurred by chance. Negative Z-score indicates that the observed connection is less than expected by chance.

INTS12 vs H3K4me3

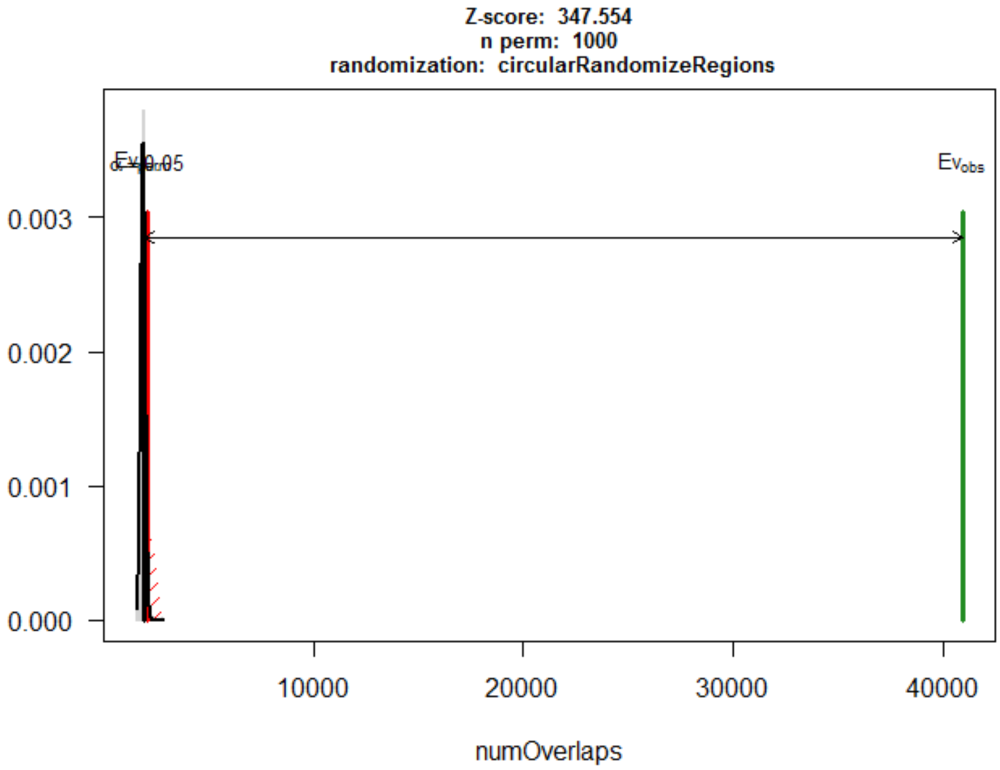

INTS12 vs H3K36me3

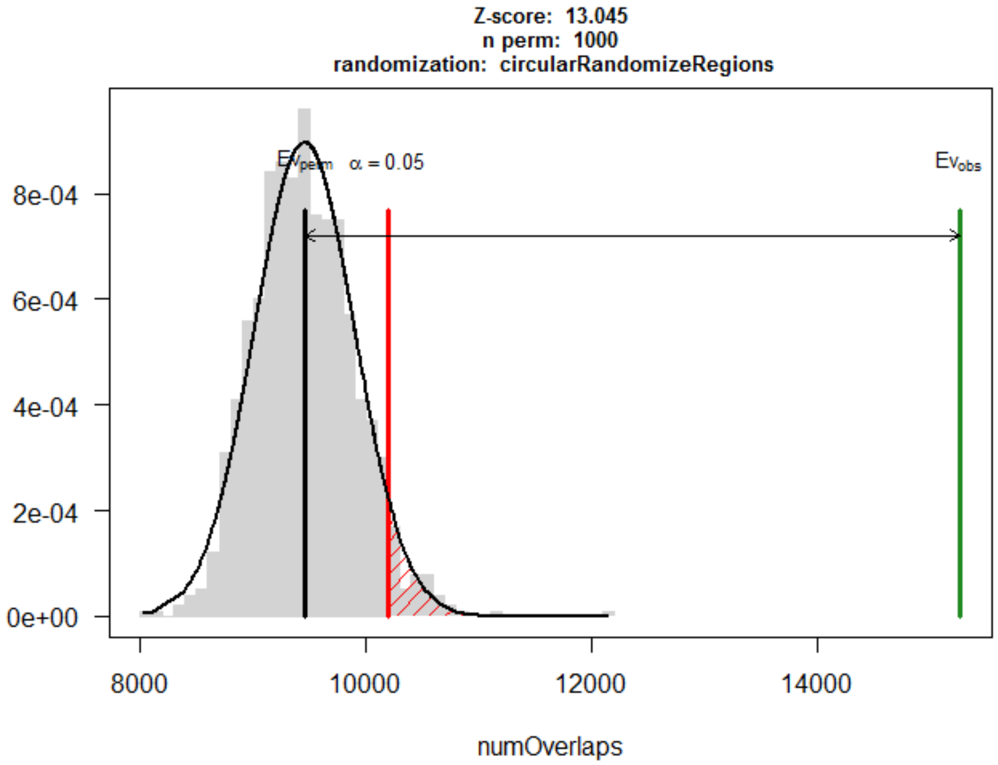

INTS12 vs H3K27me3

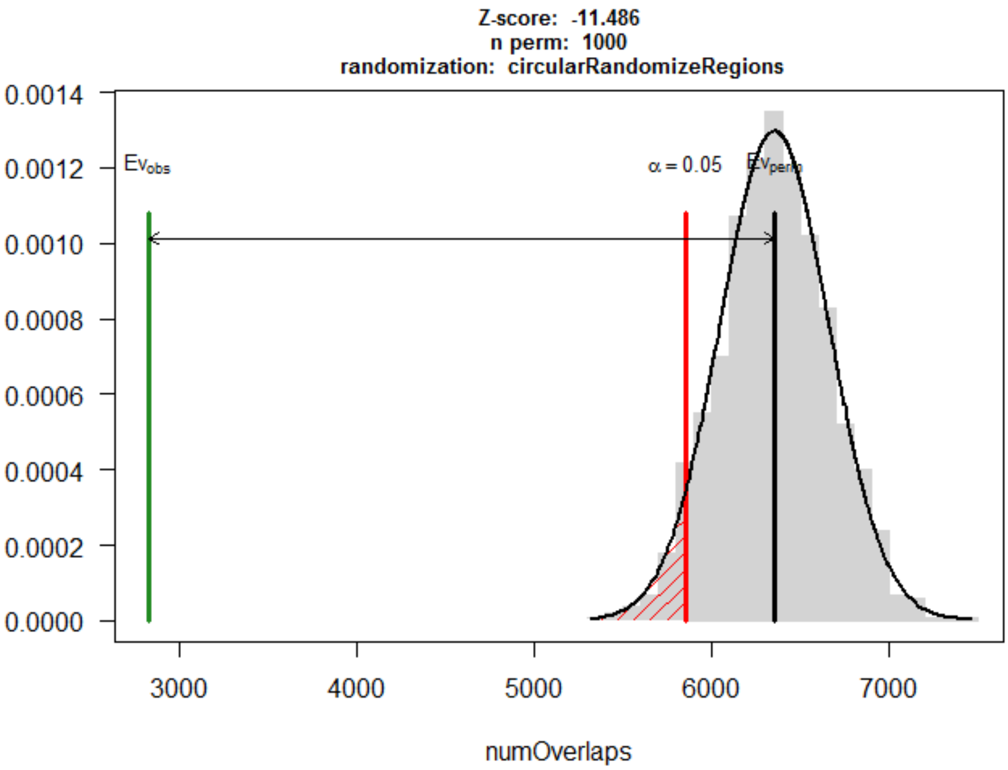

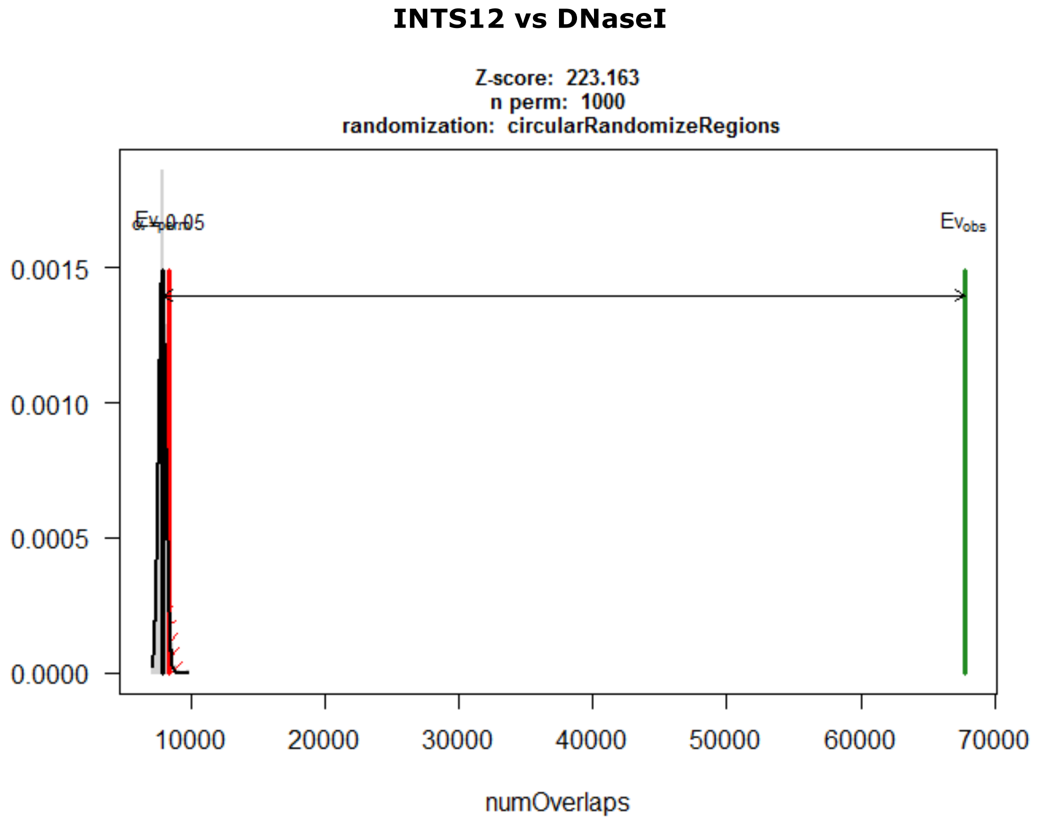
INTS12 vs DNaseI
Z-score: 223.163
n perm: 1000
randomization: circularRandomizeRegions
0.0015
0.0010
0.0005
0.0000
10000
20000
30000
40000
50000
60000
70000
numOverlaps

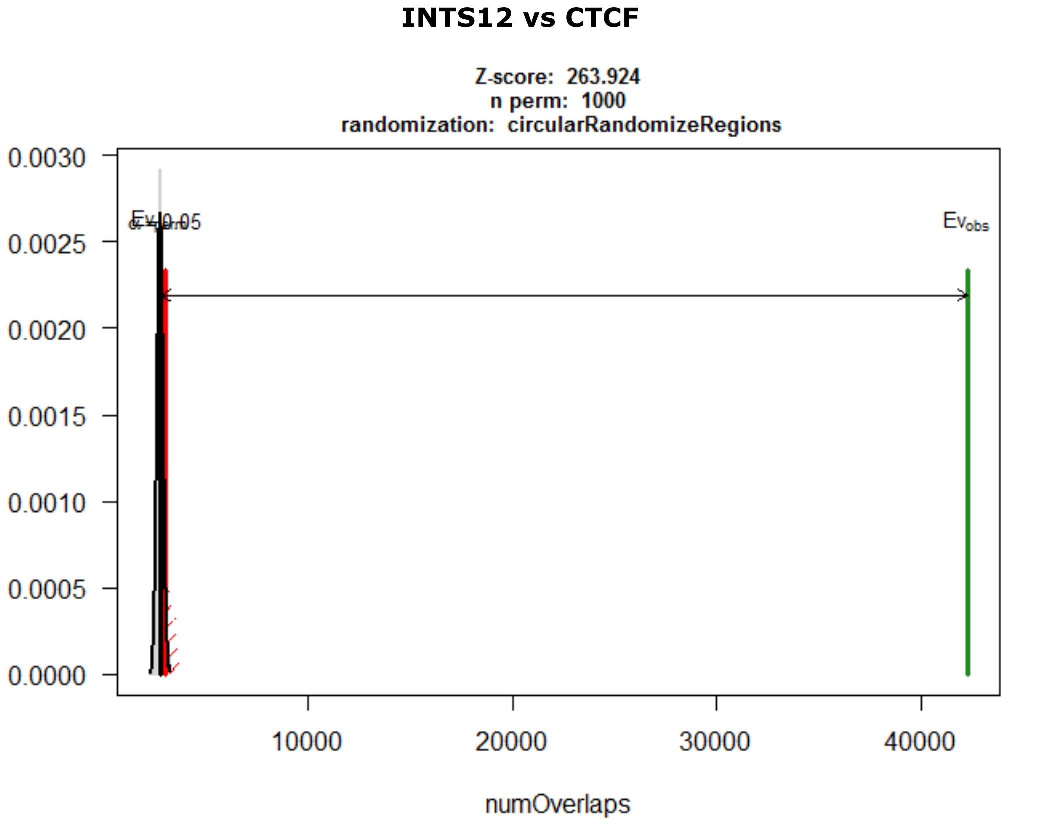
INTS12 vs CTCF
Z-score: 263.924
n perm: 1000
randomization: circularRandomizeRegions
0.0030
0.0025
0.0020
0.0015
0.0010
0.0005
0.0000
10000
20000
30000
40000
numOverlaps

Figure S12: Correlation of INTScom members at 48h and 120h. Numbers and colours are indicative of Pearson's correlation coefficients. INTS12 column is highlighted in red box next to the average of coefficients.

INTS12 appears to have poor correlation with other INTScom members in HBECs suggesting its functional independence from the rest of the complex.

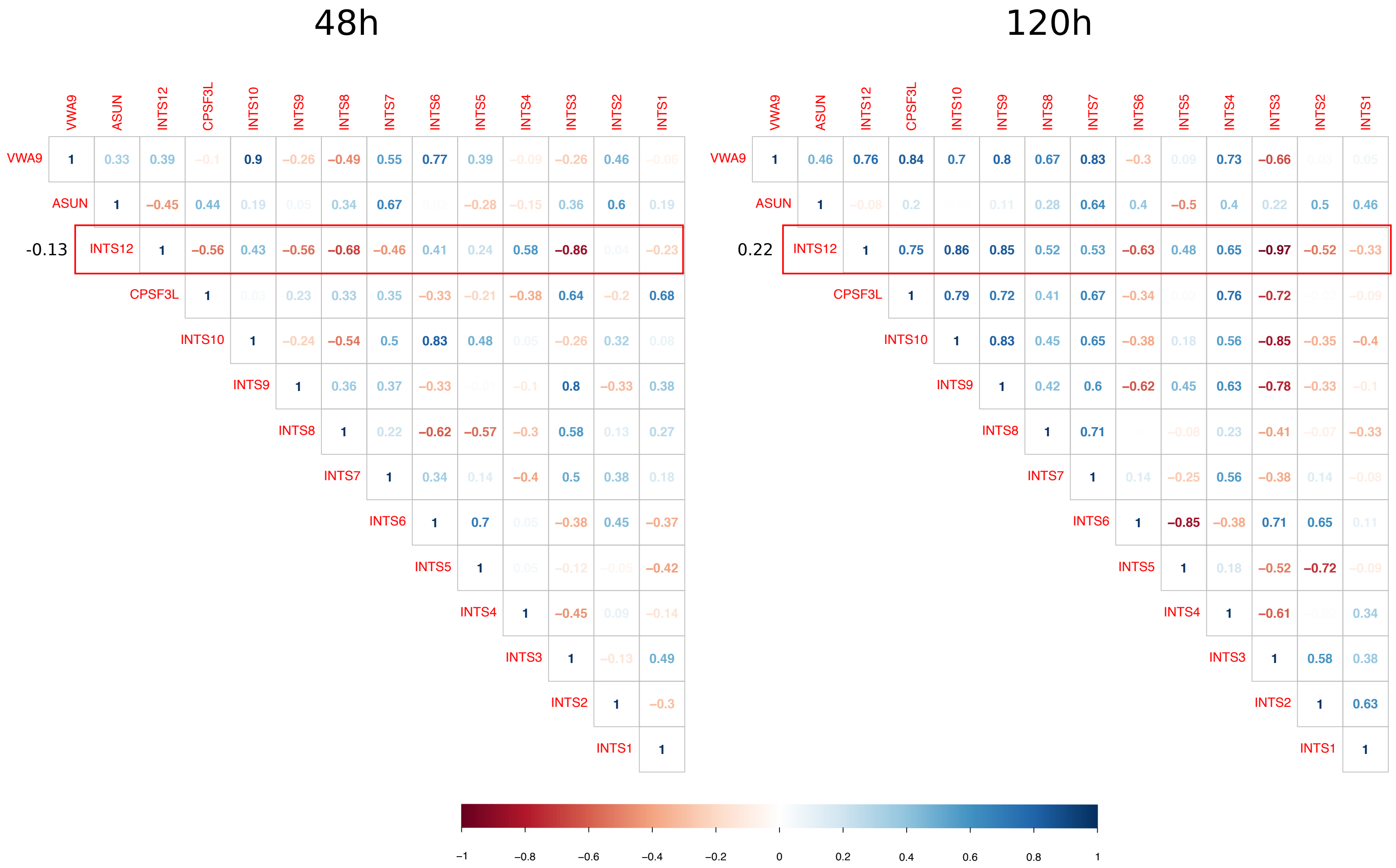

Supplement: Supplementary file 1 — Contains all supplemental figure data. Each figure has its legend. (PDF 3851 kb) [file 12864_2017_3628_MOESM1_ESM.pdf]
