# Supplementary material for: Lung function associated gene Integrator Complex subunit 12 regulates protein synthesis pathways
Source: BMC Genomics. 2017 Mar 23;18:248. doi: 10.1186/s12864-017-3628-3 (PMC5364626; doi:10.1186/s12864-017-3628-3)
Supplement: Supplementary file 2 — Contains all supplemental table data. Each table has its legend. (PDF 166 kb) [file 12864_2017_3628_MOESM2_ESM.pdf]

Supplemental Tables

Lung function associated gene Integrator Complex subunit 12 regulates protein synthesis pathways

Kheirallah et al.

Table S1: Hits of the INTS12 BLASTP search against a database of human proteins.

BLASTP search identified significant sequence similarity between canonical full length human INTS12 protein (NP_001135943.1) and PHD finger family.

BLASTP SEARCH HITS						
**Description**	**Max score**	**Total score**	**Query cover**	**E value**	**Ident**	**Accession**
PHD finger protein 1 isoform b	50.8	50.8	11%	3.00E-06	39%	NP_077084.1
PHD finger protein 1 isoform a	50.4	50.4	11%	4.00E-06	39%	NP_002627.1
PHD finger protein 21A isoform a	45.4	45.4	11%	2.00E-04	40%	NP_001095272.1
PHD finger protein 21A isoform b	44.7	44.7	14%	3.00E-04	37%	NP_057705.3
sp110 nuclear body protein isoform a	42.7	42.7	12%	0.001	40%	NP_004500.3
histone-lysine N-methyltransferase 2A isoform 1 precursor	43.1	43.1	11%	0.001	38%	NP_001184033.1
histone-lysine N-methyltransferase 2A isoform 2 precursor	43.1	43.1	11%	0.001	38%	NP_005924.2
sp110 nuclear body protein isoform c	42.7	42.7	12%	0.001	40%	NP_536349.2
metal-response element-binding transcription factor 2	41.6	41.6	11%	0.002	30%	NP_031384.1

isoform a						
metal-response element-binding transcription factor 2 isoform c	40.8	40.8	11%	0.004	30%	NP_001157863.1
metal-response element-binding transcription factor 2 isoform b	40.8	40.8	11%	0.005	30%	NP_001157864.1
bromodomain adjacent to zinc finger domain protein 2B isoform a	38.1	38.1	27%	0.034	25%	NP_038478.2
bromodomain adjacent to zinc finger domain protein 2B isoform b	38.1	38.1	27%	0.038	25%	NP_001276904.1

Table S2: Summary of proteins with sequence similarity to INTS12.

Summary of proteins showing sequence similarity to human INTS12 provide evidence for putative chromatin and gene regulation roles.

**INTS12-SIMILAR PROTEIN SUMMARIES**	
PHD finger protein 1 isoform a and b	This gene encodes a Polycomb group protein. The protein is a component of a histone H3 lysine-27 (H3K27)-specific methyltransferase complex, and functions in transcriptional repression of homeotic genes. The protein is also recruited to double-strand breaks, and reduced protein levels results in X-ray sensitivity and increased homologous recombination. Multiple transcript variants encoding different isoforms have been found for this gene. [provided by RefSeq, May 2009]
PHD finger protein 21A isoform a and b	The PHF21A gene encodes BHC80, a component of a BRAF35 (MIM 605535)/histone deacetylase (HDAC; see MIM 601241) complex (BHC) that mediates repression of neuron-specific genes through the cis-regulatory element known as repressor element-1 (RE1) or neural restrictive silencer (NRS) (Hakimi et al., 2002 [PubMed 12032298]).[supplied by OMIM, Nov 2010].
sp110 nuclear body protein isoform a and c	The nuclear body is a multiprotein complex that may have a role in the regulation of gene transcription. This gene is a member of the SP100/SP140 family of nuclear body proteins and encodes a leukocyte-specific nuclear body component. The protein can function as an activator of gene transcription and may serve as a nuclear hormone receptor coactivator. In addition, it has been suggested that the protein may play a role in ribosome biogenesis and in the induction of myeloid cell differentiation. Alternative splicing has been observed for this gene and three transcript variants, encoding distinct isoforms, have been identified. [provided by RefSeq, Jul 2008]
histone-lysine N-methyltransferase 2A isoform 1 and 2 precursor	This gene encodes a transcriptional coactivator that plays an essential role in regulating gene expression during early development and hematopoiesis. The encoded protein contains multiple conserved functional domains. One of these domains, the SET domain, is responsible for its histone H3 lysine 4 (H3K4) methyltransferase activity which mediates chromatin modifications associated with epigenetic transcriptional activation. This protein is processed by the enzyme Taspase 1 into two fragments, MLL-C and MLL-N. These fragments reassociate and further assemble into different multiprotein complexes that regulate the transcription of specific target genes, including many of the HOX genes. Multiple chromosomal translocations involving this gene are the cause of certain acute lymphoid leukemias and acute myeloid leukemias. Alternate splicing results in multiple transcript variants.[provided by RefSeq, Oct 2010]

metal-response element-binding transcription factor 2 isoform a, b and c	No description available
bromodomain adjacent to zinc finger domain protein 2B isoform a	No description available

Table S3: Sequences of D-siRNAs used for INTS12 knockdown.

Oligo	Sequence
D-siRNA #A	5′-GGAAUGGAAAUAGUGGAACAUCAGG-3′
D-siRNA #B	5′-GGCAAUCAAUUAGUAGAAUGUCAGG-3′
D-siRNA #C	5′-GCGUUUAAGAGAACAGAAGUCAAGA-3′

Table S4: Sequences of forward and reverse primers/probes used in snRNA processing and gene expression qPCR assays.

SYBR Green		
**Target**	**Oligo**	**Sequence**
Immature U1	Forward primer	5′-GATGTGCTGACCCCTGCGATTTC-3′
	Reverse primer	5′-GTCTGTTTTTGAAACTCCAGAAAGTC-3′
Immature U2	Forward primer	5′-TTGCAGTACCTCCAGGAACGG-3′
	Reverse primer	5′-CAGGGAAGCAGTTAAGTCAAGCC-3′
Immature U4	Forward primer	5′-AGCTTTGCGCAGTGGCAGTATCG-3′
	Reverse primer	5′-AGCTTTGCGCAGTGGCAGTATCG-3′
Immature U5	Forward primer	5′-TACTCTGGTTTCTCTTCAGATCGC-3′
	Reverse primer	5′-TTCTATTGTTGGATTACCAC-3′
MARS	Forward primer	5′-TACCCATTACTGCAAGATCC-3′
	Reverse primer	5′-CTTGCTGTTTCAGTACAGTC-3′
GARS	Forward primer	5′-GTGTTAGTGGTCTGTATGAC-3′
	Reverse primer	5′-GTCTTTAAAACTGGCTCAGG-3′
ASNS	Forward primer	5′-GATTGGCTGCCTTTTATCAG-3′
	Reverse primer	5′-AATTGCAAATGTCTGGAGAG-3′
ATF4	Forward primer	5′-CCTAGGTCTCTTAGATGATTACC-3′
	Reverse primer	5′-CAAGTCGAACTCCTTCAAATC-3′
LEP	Forward primer	5′-TCAATGACATTTCACACACG-3′
	Reverse primer	5′-TCCATCTTGGATAAGGTCAG-3′
**TaqMan**		
INTS12	Forward primer	5′-CTCCAGCTGTCAAAGATCCATT-3′
	Reverse primer	5′-GAGAGCTGCTGGATTCTGAAGT-3′
	Probe	5′-TGGCTGCAAAAGCTGCCCATCCAG-3′

Table S5: Sequences of forward and reverse primers used in ChIP-PCR assays of INTS12 binding to the indicated sites.

Target gene/primer	Primer sequence	Site (hg19)	Type of binding
POR forward	5′-CAGGGTCCGAGCTGTAGAAG-3′	TSS-145	Positive
POR reverse	5′-CCGGCAGAGAAATGAAAGTG-3′		
NBPF1 forward	5′-CACCTACGCCTCCCAGTACC-3′	TSS+108	Positive
NBPF1 reverse	5′-GCCTTGGGTTATCCTGACAC-3′		
ACTB forward	5′-AACTCTCCCTCCTCCTCTTCC-3′	TSS-154	Positive
ACTB reverse	5′-CCTCTCCCCTCCTTTTGC-3′		
Untr12 forward	5′-TGAGCATTCCAGTGATTTATTG-3′	Chr12:61667747–61667824	Negative
Untr12 reverse	5′-AAGCAGGTAAAGGTCCATATTTC-3′		

Table S6: A summary of the survey of scientific literature that investigated the effect of INTScom members on snRNA processing. Ezzedine et al. 2011, Chen et al. 2012 and Chen et al. 2013 studies were undertaken on fly S2 cells while Baillat et al. 2005 study was undertaken on human HeLa cells.

Study	INTScom protein targeted	Effect on snRNA processing
Ezzedine et al. 2011	INTS1	Moderate
Ezzedine et al. 2011	INTS2	Strong
Ezzedine et al. 2011	INTS3	Very weak
Ezzedine et al. 2011	INTS4	Very strong
Ezzedine et al. 2011	INTS5	Moderate
Ezzedine et al. 2011	INTS6	Weak
Ezzedine et al. 2011	INTS7	Weak
Ezzedine et al. 2011	INTS8	Moderate
Ezzedine et al. 2011	INTS9	Very strong (two experiments)
Ezzedine et al. 2011	INTS10	Very weak
Ezzedine et al. 2011	INTS11	Strong
Ezzedine et al. 2011	INTS12	Very weak (one experiment) and weak (another experiment)
Ezzedine et al. 2011	INTS1	Moderate
Ezzedine et al. 2011	INTS2	Strong
Ezzedine et al. 2011	INTS3	Very weak
Ezzedine et al. 2011	INTS4	Very strong
Ezzedine et al. 2011	INTS5	Moderate
Ezzedine et al. 2011	INTS6	Weak
Ezzedine et al. 2011	INTS7	Weak
Ezzedine et al. 2011	INTS8	Moderate
Ezzedine et al. 2011	INTS9	Very strong (two experiments)
Ezzedine et al. 2011	INTS10	Very weak
Ezzedine et al. 2011	INTS11	Strong
Ezzedine et al. 2011	INTS12	Very weak (one experiment) and weak (another experiment)
Ezzedine et al. 2011	INTS1	Above 30 and below 100 fold relative to control

Ezzedine et al. 2011	INTS2	Below 3 fold relative to control
Ezzedine et al. 2011	INTS3	Less than 1 fold relative to control
Ezzedine et al. 2011	INTS4	300 fold relative to control
Ezzedine et al. 2011	INTS5	Below 3 fold relative to control
Ezzedine et al. 2011	INTS6	Below 3 fold relative to control
Ezzedine et al. 2011	INTS7	10 fold relative to control
Ezzedine et al. 2011	INTS8	Below 3 fold relative to control
Ezzedine et al. 2011	INTS9	Above 100 but below 300 fold relative to control
Ezzedine et al. 2011	INTS10	Below 3 fold relative to control
Ezzedine et al. 2011	INTS11	Above 3 but below 10 fold relative to control
Ezzedine et al. 2011	INTS12	Below 3 fold relative to control
Ezzedine et al. 2011	INTS1	Above 30 and below 100 fold relative to control
Ezzedine et al. 2011	INTS2	Above 3 but below 10 fold relative to control
Ezzedine et al. 2011	INTS3	Less than 1 fold relative to control
Ezzedine et al. 2011	INTS4	Above 300 and below 1000 fold relative to control
Ezzedine et al. 2011	INTS5	Above 10 fold relative to control
Ezzedine et al. 2011	INTS6	Above 3 but below 10 fold relative to control
Ezzedine et al. 2011	INTS7	10 fold relative to control
Ezzedine et al. 2011	INTS8	Above 3 but below 10 fold relative to control
Ezzedine et al. 2011	INTS9	Above 100 but below 300 fold relative to control
Ezzedine et al. 2011	INTS10	Below 3 fold relative to control
Ezzedine et al. 2011	INTS11	Above 10 but below 30 fold relative to control
Ezzedine et al. 2011	INTS12	Above 3 but below 10 fold relative to control
Ezzedine et al. 2011	INTS1	30 fold relative to control

Ezzedine et al. 2011	INTS2	Below 3 fold relative to control
Ezzedine et al. 2011	INTS3	Less than 1 fold relative to control
Ezzedine et al. 2011	INTS4	Above 30 and below 100 fold relative to control
Ezzedine et al. 2011	INTS5	3 fold relative to control
Ezzedine et al. 2011	INTS6	Below 3 fold relative to control
Ezzedine et al. 2011	INTS7	10 fold relative to control
Ezzedine et al. 2011	INTS8	Below 3 fold relative to control
Ezzedine et al. 2011	INTS9	30 fold relative to control
Ezzedine et al. 2011	INTS10	Below 3 fold relative to control
Ezzedine et al. 2011	INTS11	3 fold relative to control
Ezzedine et al. 2011	INTS12	Below 3 fold relative to control
Ezzedine et al. 2011	INTS1	Above 30 and below 100 fold relative to control
Ezzedine et al. 2011	INTS2	Above 3 but below 10 fold relative to control
Ezzedine et al. 2011	INTS3	Below 3 fold relative to control
Ezzedine et al. 2011	INTS4	300 fold relative to control
Ezzedine et al. 2011	INTS5	Above 10 fold relative to control
Ezzedine et al. 2011	INTS6	Above 3 but below 10 fold relative to control
Ezzedine et al. 2011	INTS7	Above 10 but below 30 fold relative to control
Ezzedine et al. 2011	INTS8	Above 3 but below 10 fold relative to control
Ezzedine et al. 2011	INTS9	100 fold relative to control
Ezzedine et al. 2011	INTS10	Below 3 fold relative to control
Ezzedine et al. 2011	INTS11	Above 3 but below 10 fold relative to control
Ezzedine et al. 2011	INTS12	3 fold relative to control
Chen et al 2012	INTS12	Moderate
Chen et al 2012	INTS12	Moderate
Chen et al 2012	INTS9	Above 30 but below 100 fold relative to

		control
Chen et al 2012	INTS9	Above 10 but below 30 fold relative to control
Chen et al 2012	INTS12	Above 1 but below 3 fold relative to control
Chen et al 2012	INTS12	Above 1 but below 3 fold relative to control
Chen et al 2013	INTS12	Moderate
Chen et al 2013	INTS12	Moderate
Chen et al 2013	INTS12	Between 5 to 7 fold relative to control
Chen et al 2013	INTS12	Between 1 to 3 fold relative to control
Chen et al 2013	INTS12	Between 3 to 5 fold relative to control
Chen et al 2013	INTS12	Between 3 to 5 fold relative to control
Chen et al 2013	INTS12	No effect
Baillat et al. 2005	INTS11	3 fold relative to control
Baillat et al. 2005	INTS11	4 fold relative to control
Baillat et al. 2005	INTS1	2 fold relative to control
Baillat et al. 2005	INTS1	4 fold relative to control
