# Supplementary material for: Lung function associated gene Integrator Complex subunit 12 regulates protein synthesis pathways
Source: BMC Genomics. 2017 Mar 23;18:248. doi: 10.1186/s12864-017-3628-3 (PMC5364626; doi:10.1186/s12864-017-3628-3)
Supplement: Supplementary file 3 — Contains a more detailed supplemental information in relation to the methods: Cell Culture, RNAi, RNAseq, qPCR, RNAseq and Pathway Data Analysis [63], Protein synthesis by 35S-Methionine incorporation assay [64], Assessment of proliferative capacity by cell counts, ChIPseq, ChIP-PCR, ChIPseq Data Analysis [65–68], ENCODE data retrieval and analysis [69], Immunofluorescence [70]. (PDF 89 kb) [file 12864_2017_3628_MOESM3_ESM.pdf]

Supplemental Methods

Lung function associated gene Integrator Complex subunit 12 regulates protein synthesis pathways

Kheirallah et al.

Cell Culture

Human bronchial epithelial cells (HBEC) were purchased from Clonetics-Biowhittaker (MD, USA). Cells were cultured in HBEC basal medium (BEGM) from Lonza (Berkshire, UK; Product code CC-2540) prepared by addition of all the recommended supplements per manufacturer specifications excluding gentamicin. All laboratory experiments were performed using passage 3 cells. Prior to experiments cells were grown at 37°C with 5% CO_2 until ~95% confluent with BEGM media change every ~48h.

RNAi

Interferin (Polyplus Transfection) was used for gene knockdown optimizations. INTS12 silencing efficiency was tested using D-siRNAs A, B and C (OriGene, Table S3). Subsequently D-siRNAs A and C were tested at 0.1nM, 1nM and 10nM concentrations and a concentration of 1nM was chosen for optimal silencing efficiency. Two D-siRNAs were used in the experiments to account for off-target effects and thus to internally validate our observations. For main RNAseq and functional experiments the effects of INTS12 depletion were assessed 120h after initiation of interference. To ensure appropriate knockdown D-siRNA transfections were administered on two occasions at days zero and three of the experiment. To compare the acute and chronic transcriptomic responses to knockdown, RNAseq profiling was also performed 48h after the initiation of interference. In all experiments there were four experimental conditions: un-transfected cells, cells transfected with scrambled D-siRNA control, and cells transfected with D-siRNAs A and C. Each experimental condition was performed in three independent biological replicates.

RNAseq

Total RNA was extracted using a mammalian total RNA prep kit with on-column DNaseI digestion (Sigma-Aldrich). Sequencing samples were ensured to have RNA integrity number scores greater or equal to 8 (Agilent Technologies). The sequencing libraries were prepared with Illumina TruSeq RNA Sample Prep Kit v2. mRNA was poly-A selected by capturing total RNA samples with oligo-dT coated magnetic beads. The mRNA was then fragmented and randomly primed. cDNA was synthesised using random primers. Finally, a ready-for-sequencing library was prepared by end-repair, phosphorylation, A-tailing, adapter ligation and PCR amplification. Paired-end sequencing was performed on the HiSeq2000 platform (Illumina) using TruSeq v3 chemistry over 100 cycles yielding approx. 40 million reads per sample.

qPCR

Cultured cells were lysed and RNA was extracted using silica-membrane columns (Sigma-Aldrich). 1µg of total RNA was converted to cDNA using the SuperScript synthesis system leveraging random hexamer priming (Invitrogen). Prior to reverse transcription, RNA was treated with DNaseI for a second time to ensure complete removal of any remaining traces of genomic DNA (gDNA). Each reverse transcriptase positive sample had equivalent reverse transcriptase negative control sample. For TaqMan assays (Applied Biosystems) the final volume of qPCR mix per single well was 20µl consisting of 2µl of cDNA template, 6.4µl of DNase and RNase free water, 0.3µM of forward primer, 0.3µM of reverse primer, 0.1µM of probe, and 10µl of x2 TaqMan master mix (Applied Biosystems). For SYBR Green assays the final volume of qPCR mix per single well was 25µl consisting of 5µl of cDNA template, 6.4µl of DNase and RNase free water, 0.25µM of forward primer, 0.25µM of reverse primer, and 12.5µl of x2 Brilliant III Ultra-Fast SYBR Green master mix (Agilent). Reverse transcriptase positive samples were run in triplicate while reverse transcriptase negative samples were run in duplicates. Every qPCR ran had a water only control. qPCR oligo sequences are shown in the Table S4. Housekeeping GAPDH expression was run using pre-developed assay reagents (Life Technologies). QPCR-derived relative to GAPDH and control gene expression was analysed using ΔΔCt method [62]. QPCR technical validation of RNAseq findings was performed using at least three biological cDNA replicates derived from total RNA used in sequencing thus were upon the same donor cells. QPCR biological validation of target genes was performed upon different donor cells with at least three biological cDNA replicates.

RNAseq and Pathway Data Analysis

The quality of raw fastq files was assessed on fastqc. Tuxedo analysis pipeline was used for RNAseq analysis [63]: (1) TopHat read alignment was performed upon hg19 build, (2) Cufflinks transcriptome assembly was performed on individual sample basis and merged by Cuffmerge using reference-based assembly, (3) Cuffdiff differential gene expression was performed using Cuffmerge-predicted annotation. Loci with Benjamin-Hochberg corrected P value [60] below 0.05 were considered significant. Transcriptomic comparisons were performed comparing scrambled D-siRNA to each anti-INTS12 D-siRNA and comparing un-transfected cells with scrambled D-siRNA transfected cells in order to account for off-target and mere transfection effects respectively.

In order to perform pathway analyses, fragments per kilobase per million reads (FPKM) expression values were obtained for each gene per individual RNAseq sample using Cuffnorm. Loci containing multiple amalgamated genes were separated into individual genes and had assigned the equivalent expression values, while genes occurring multiple times on the dataset had their expression values summated using in-house written python script. Scripts can be accessed on GitHub repository (https://github.com/msxakk89/dataset_preperation_scripts). Gene set enrichment analysis using 4722 curated gene sets including 1320 canonical pathway definitions from the Molecular Signatures Database [35] was used, comparing scrambled D-siRNA to each anti-INTS12 D-siRNA and comparing un-transfected cells with scrambled D-siRNA transfected cells. Pathways with Benjamin-Hochberg corrected P value below 0.05 were considered significant. Pathways reproducibly dysregulated by the two different D-siRNA treatments were considered further. Top candidate pathways with the highest enrichment score in both D-siRNAs were chosen for further functional analysis. Results of the pathway analysis were displayed in a Cleveland's plot using ggplot2 R package while pathway heatmaps were drawn using heatplus R package. Boxplots were drawn using build-in R function. Pearson's correlations of gene expression were calculated using hmisc R package and drawn using ggplot2.

Comparison of acute and chronic transcriptomic responses to INTS12 knockdown aimed at identifying core subset genes significantly differentially expressed in 48h and 120h time points respectively. The rational of the analysis was similar to pathway analysis, i.e. genes were shortlisted if were reproducibly dysregulated in both anti-INTS12 D-siRNAs but not in scrambled D-siRNA. Genes that were dysregulated in both anti-INTS12 D-siRNAs in a given direction while in the opposite direction in the scrambled D-siRNA sample were also included.

Core subset of genes was identified by determining the common genes between the 48h and 120h significant gene lists. Enrichment of lung biology relevant gene set was performed via Fisher's exact over-representation analysis using the background of protein coding genes. Correlation of INTS12 with INTScom was calculated by averaged Pearson's correlation over all the complex members.

Protein synthesis by ^{35}S-Methionine incorporation assay

Rates of protein synthesis were measured using EasyTag ^{35}S protein labelling for 10 minutes in labelling medium, followed by lysis of cells in passive lysis buffer (Promega) and TCA precipitation on filter paper as described previously for NIH3T3 cells [64]. Three biological replicates with four technical replicates each were performed. In parallel, the same samples were assayed for total protein using 200µl Coomassie Protein Assay Reagent (Thermo) with 10µl of lysate in microtitre plates and a Synergy HT plate reader (Biotek) at 595 nm. Background for lysis buffer alone was subtracted. For each replicate, the radioactive incorporation was divided by the protein assay measurements thus yielding a measure of incorporation per amount of total protein. Statistical significance of difference in protein synthesis in INTS12 depleted cells was determined by one-way ANOVA analysis of variance followed by Fisher's Least Significant Difference test.

Assessment of proliferative capacity by cell counts

Proliferative capacity was assessed by comparing total cell counts at the beginning and at end of the knockdown, i.e. at the beginning of experiment cells were seeded at the same density in all the conditions. At the end HBECs were washed with PBS, treated with trypsin/EDTA at 37°C for 10min to allow all the cells to detach and were re-suspended in 1ml of culture media. Samples were coded and mixed to perform counting without knowledge of the condition and conditions were decoded later. Cell counts were performed on haemocytometer in technical triplicate per each condition, averaged and total cell count estimates derived accordingly. Experiment was performed in four biological replicates.

ChIPseq

HBECs from two different donors were fixed with formaldehyde solution for 15 min. Formaldehyde solution contained 11% formaldehyde (Sigma), 0.1M sodium chloride (Sigma), 1mM EDTA (Sigma), 50mM 4-(2-hydroxyethyl)-1-piperazineethanesulfonic acid (Applichem). Fixation was quenched with 0.125 M glycine (Sigma). Chromatin was isolated by the addition of lysis buffer (Active Motif), followed by disruption with a Dounce homogenizer (Active Motif) to allow for efficient chromatin preparation. Lysates were sonicated and the DNA sheared to an average length of 300-500bp. Genomic DNA for each replicate sample was prepared by treating aliquots of chromatin with RNase, proteinase K and heat for de-crosslinking, followed by ethanol precipitation (Active Motif). Pellets were re-suspended and the resulting DNA was quantified on a NanoDrop spectrophotometer. Extrapolation to the original chromatin volume allowed quantitation of the total chromatin yield. 30µg chromatin of each sample was precleared with protein A agarose beads (Invitrogen). Unprecipitated genomic DNA (i.e. input control) was prepared from a pool of equal aliquots of the two donor samples. Genomic DNA regions of interest were isolated using 4µg of antibody against INTS12 (Sigma cat. num. HPA03577) following manufacturer's specifications (Active Motif). Complexes were washed, eluted from the beads with SDS buffer, and subjected to RNase and proteinase K treatment. Crosslinks were reversed by incubation overnight at 65°C, and ChIP DNA was purified by phenol-chloroform extraction and ethanol precipitation. Initially a pilot experiment was conducted where DNA libraries obtained from single donor were sequenced on NextSeq 500 sequencing machine (Illumina) yielding ~8 million single-ended 75bp reads in order to assess the success of ChIPseq. For the definite experiment, sequencing libraries (Illumina) were prepared from the both ChIP and input DNAs and the resulting libraries were sequenced yielding ~40 million reads per two ChIP samples from each donor cells and one input control of both donors.

ChIP-PCR

INTS12 peak regions used for qPCR validation were prioritized based on ChIPseq signals observed on the genome browser. Three positive regions and one negative region were chosen for ChIP-PCR validation. PCR primers were designed to span these regions (Table S5). qPCR reactions were carried out in triplicate upon 12.5ng of gDNA from each donor and input control using SYBR Green assay (Bio-Rad). Ct values were converted into the number of binding events detected per 1000 cells according to the manufacturers of ChIP-PCR kit specifications (Active Motif).

ChIPseq Data Analysis

Reads were BWA aligned [65] to hg19 using default settings. Artefactual read duplicates were removed using samtool prior to further analyses. MACS INTS12 peak calling was run on each donor separately comparing ChIPseq samples to input control [66]. Calling was performed with a multiple comparisons corrected P value of less than 0.05 considered as significant. Generated fragment pileup signal was normalized to library size. Fragment pileup was converted to wig files based on fold enrichment above input background for each donor. To compare peak metrics between two donor samples, overlapping intervals were grouped into active regions, which were defined by the start coordinate of the most upstream interval and the end coordinate of the most downstream interval. In locations where only one sample had an interval, this interval defined the active region. ChIP signal at these active regions was compared between the two donor samples and correlation drawn and calculated by ggplot2 and rcmdr R packages respectively. Intervals were annotated, percentage of total INTS12 binding sites falling on the fixed annotated genomic features and enrichment over meta-gene body determined using CEAS package [67]. The proportion of binding proximal to TSS was calculated by dividing the number of significant peaks close the TSS (TSS±1000bp) by the number of significant peaks falling within the broader region surrounding the TSS (TSS±3000). Enrichment over various gene classes, expressed/not expressed, or differentially expressed genes was drawn using ngs.plot [68]. Gene classes were retrieved using Ensembl's BioMart tool. HOMER and MEME were used for *de novo* identification of enriched DNA motif at INTS12 binding sites [51, 52]. TomTom was used to compare *de novo* identified motif to a set of currently known motifs [53]. BETA was used to predict INTS12 regulatory function [49].

ENCODE data retrieval and analysis

Airway epithelial cells specific epigenetic and CTCF ChIPseq datasets were obtained from ENCODE data repository (ENCBS417ENC; www.encodeproject.org) and analysed as INTS12 ChIPseq datasets with the only difference that broad region calling was used for the epigenetic marks. Percent of overlap between INTS12 intervals and ENCODE intervals and its statistical significance was determined using regioneR R package with random permutation test. Correlation of ChIPseq signals and conservation of binding analyses were performed using cistrome [69].

Immunofluorescence

Cells were grown on 8-chamber glass slides seeding 8000 cells onto each chamber and were left un-treated or were transfected with anti-INTS12 and scrambled D-siRNAs as described previously. Cells were fixed using 4% formaldehyde and blocked/permeabilized with PBS, 10% goat serum, 1% BSA, and 0.15% Triton-X. Cells were incubated with antibody against INTS12 (Sigma cat. num. HPA03577) at 4°C overnight and rhodamine-TRITC labelled secondary for 1 hour at room temperature. Controls were incubated with primary isotype control (Abcam) antibody followed by secondary antibody. Cells were visualized epifluorescently and exposures were kept constant across the conditions to avoid artefactual differences in the observed fluorescence intensity.
